# Genetic Architecture of Grain Protein Content in Hulless Barley: Insights from Multi-Environment GWAS

**DOI:** 10.3390/plants15152304

**Published:** 2026-07-27

**Authors:** Sadaf Memon, Rizwan Ali Kumbhar, Shabana Memon, Shah Nawaz Mari Baloch, Rania Chourouk Benhafid, Kehan Yang, Zebaman, Longfei Zeng, Cile Duoji, Qiji Zhuoma, Mingxiang Wang, Mingyong, Xiaoqin Yang, Yajie Liu, Hui Zhao, Zongyun Feng

**Affiliations:** 1 College of Agronomy/State Key Laboratory of Crop Gene Exploration and Utilization in Southwest China, Sichuan Agricultural University, Chengdu 611130, China; sadafmemon25@gmail.com (S.M.); rizwankunbhar@gmail.com (R.A.K.); rc.benhafid@gmail.com (R.C.B.); yangkehan0126@163.com (K.Y.); 13778699657@163.com (Z.); 18879079535@163.com (L.Z.); liuyajie@sicau.edu.cn (Y.L.); 2 Department of Plant Breeding and Genetics, Sindh Agriculture University, Tandojam 70060, Pakistan; sheeba.memon786@gmail.com (S.M.); snmari@sau.edu.pk (S.N.M.B.); 3 Luhuo County Agriculture, Rural Affairs and Science and Technology Bureau, Luhuo 626500, China; 18283647671@163.com (C.D.); 18942838607@163.com (Q.Z.); 4 Sertar County Agriculture, Rural Affairs and Science and Technology Bureau, Sertar 626600, China; 15181213555@163.com; 5 Bureau of Science, Technology, Agriculture, Animal Husbandry and Water Affairs of Aba County, Aba 624600, China; 13698172719@163.com (M.); abxngb@163.com (X.Y.)

**Keywords:** hulless barley, grain protein content, GWAS, multi-environment, SNP markers, candidate genes

## Abstract

Hulless barley (HB) is a nutrient-rich cereal gaining renewed research interest in the Tibetan regions of China. Grain protein content (GPC) is a quality-determining trait in HB that significantly affects end-use quality and is strongly influenced by environmental factors. Identifying stable association regions and developing breeding-applicable molecular markers are breeding objectives for high-GPC HB variety improvement. This study analyzed 266 HB accessions grown across six environments (2018–2021). Phenotypic GPC ranged from 5.02% to 18.31%, with moderate heritability (*H*^2^ = 0.473) and significant G×E interaction. A genome-wide association study (GWAS) using 168,983 GBS-derived SNPs and three models (GLM, MLM, FarmCPU) revealed no Bonferroni-significant SNPs in the BLUP meta-analysis. At the suggestive threshold (*p* < 1 × 10^−5^), 40 SNPs (18 loci) showed nominal association, with chromosome 7H harboring the most robust cross-model signal. Two SNPs on chromosome 6H and 7H exceeded Bonferroni correction in Yangma-2018 under GLM and FarmCPU. Candidate gene mining revealed seven functional genes, including 30S ribosomal protein S13 and glutamate-cysteine ligase. The lead SNP S7H_6966377 was located 165 kb from 30S ribosomal protein S13. These findings establish chromosome 7H as a regulatory hub for GPC and provide markers for marker-assisted selection.

## 1. Introduction

Barley (*Hordeum vulgare* L.) is a cool-season annual cereal crop that plays a key role in agriculture, specifically as a cover crop [1]. It is categorized as an ideal cereal crop due to its diploid genome, low basic chromosome number (*n* = 7), and membership in the *Tritaceae* tribe alongside wheat, through the species diverged approximately 8–15 million years ago [2]. Barley exhibits extensive genetic diversity, with minimal reproductive barriers separating cultivated forms from wild ancestors [3,4]. Additionally, barley domestication dates back to the early agricultural revolutions, approximately 8000 to 10,000 years ago in the Fertile Crescent [5,6]. It ranks as the fourth most valued cereal after wheat, rice, and maize [7], and it is the fifth most widely grown crop globally [8]. Barley was introduced to China approximately 5200 years ago and to the Tibet Plateau around 3600 years ago [9,10,11]. Globally, around 75% of barley is consumed as animal feed, 20% is used for malting and beverages production, and only 5% is utilized for human food [12,13].

Hulless barley (*Hordeum vulgare* L. var. *nudum* Hook. f.; hereafter HB) is a six-rowed, naked caryopsis cultivar valued for its nutrient profile. Unlike hulled barley, HB can be processed without removing the hull, resulting in higher concentrations of protein, starch, and β-glucan [14,15]. It is predominantly cultivated in the Qinghai–Tibet Plateau, spanning regions like Tibet, Qinghai, Gansu, Sichuan, and Yunnan in China [16]. HB exhibits broad adaptability compared to other cereal crops, characterized by its resistance to cold and drought, tolerance to poor alkaline soils, robust stress tolerance, and a relatively short growth cycle, positioning it as a significant subject for scientific study [3,17]. These attributes, combined with its cold-hardness [18,19,20], and significant genetic variation as a short-life, self-pollinating species, position HB as an ideal candidate for hybridization and agricultural development [21,22].

### 1.1. Grain Protein Content (GPC): Nutritional Significance and Quality Parameters

Barley grain proteins are classified into two main groups: seed storage proteins and structural proteins. Storage proteins, primarily hordeins, accumulate in the endosperm during grain-filling phase and constitute 35–55% of total protein content [23,24]. Structural proteins, which including albumins, globulins, and amphiphilic proteins, also contribute to the grain’s overall protein profile [25,26]. Protein accounts for approximately 8–27% of the total grain weight and is essential for storage, structural, and metabolic functions, while, carbohydrates—primarily starch, sugars, and non-starch polysaccharides—represent roughly 80% of dry grain weight [27]. The intricate relationship between protein content and grain quality depends strongly on intended end-use: high protein content is desirable for feed applications as it enhances the nutritional value [28], whereas moderate protein with high starch is preferred for malting and brewing [29].

HB has garnered attention for its superior nutritional value compared to hulled varieties, particularly its elevated protein content, making it well-suited for diverse food and feed applications [30]. Barley is rich in bioactive phytochemicals—including β-glucans, phenolics, tocols, and phytosterols—that confer significant health advantages [31,32], such as enhanced antioxidant effects and improved regulation of chronic metabolic and cardiovascular conditions. Its low fat, high starch, protein, and fiber content (both soluble and insoluble) offer specific health benefits [33,34]. The high protein content of HB is particularly valuable in animal feed [35] and increasingly in human consumption food products, including breakfast cereals, baby food, and bakery items.

Furthermore, hulled barley is the primary typed utilized in brewing as malt for alcoholic beverages, while HB is predominantly consumed as a whole grain flour [36]. The inclusion of protein-rich cereal-based products in the diet is recognized as a key factor in promoting human health and preventing various diseases and cancers [37]. Both hulled and HB provide strong antioxidant benefits and maintain a low glycemic index, which play vital roles in reducing the risk of chronic diseases; however, hulled barley demonstrates superior nutritional quality through higher crude protein concentration and lower crude fiber content [34,38]. Additionally, HB grain contains various phytochemicals, including sterols, tocopherols, tocotrienols, phenolic compounds, and antioxidants [39]. In brief, barley is an active source of starch, minerals, vitamins, and proteins, making it a valuable health food supplement [40].

GPC in modern barley cultivars typically ranges between 8% and 14%, depending on fertilization rates [41]. HB has been reported to exhibit a protein content of 14.23%, compared to 12.35% in hulled barley [42]. Historically, barley GPC has been documented to range from 5% and 18%, averaging approximately 12% [43]. Optimal protein requirements vary substantially by end-use. For livestock feed, a protein content of 14% to 15.5% is considered optimal [44] whereas for malting and brewing, the desirable protein content is approximately 12% and should not exceed 13.5%. For grain distillers, protein content in two-rowed barley ranges from 11.5% to 14%; six-rowed barley, which includes the hulless type, typically contains 12% to 13.5% protein and is primarily used for food and feed rather than malting [45,46]. Given the central importance of GPC in determining end-use quality and nutritional value, understanding the genetic architecture underlying GPC variation in HB is essential for targeted breeding improvement.

### 1.2. Background of the Study

HB was cultivated across six diverse environments in Sichuan, China—Yangma (2018, 2019, 2021), Deyang (2019), Nanchong (2019), and Kangding (2020)—to capture broad phenotypic variation in GPC. While three-year phenotypic data from each location is ideal for multi-environment genome-wide association study (GWAS), the current design leverages multi-location evaluation across contrasting agro-ecological zones (Sichuan Basin vs. Tibetan Plateau) to maximize genotype-by-environment (G×E) interaction capture. The best linear unbiased prediction (BLUP) analysis accounts for environment as a fixed effect and genotype as a random effect, effectively handling unequal replication across environments [47]. Sensitivity analysis using Yangma-only data (three years) yielded comparable association signals, supporting the validity of the multi-location approach for stable quantitative trait locus (QTL) detection. Both genetic and environmental factors are known to significantly influence GPC; however, the genetic architecture underlying this trait in HB remains poorly understood. To explore this trait, we designed a layout of the current study for the assessment of phenotypic variation across six environments, and by integrating phenotypic and genotyping-by-sequencing (GBS) data, GWAS was performed to identify significant quantitative trait nucleotides (QTNs) associated with GPC and mine seven core related candidate genes.

Though in-depth analysis of GWAS-based single nucleotide polymorphisms (SNPs), several stable QTNs were detected in genomic hotspot regions. These findings provide a foundation for markers-assisted selection (MAS) in breeding programs. This research enhances understanding of the genetic basis of GPC in HB and supports the development of breeding strategies to improve both the nutritional quality and market value of this crop.

## 2. Results

### 2.1. Phenotypic Variation and Environmental Stability of GPC

**Figure 1:**
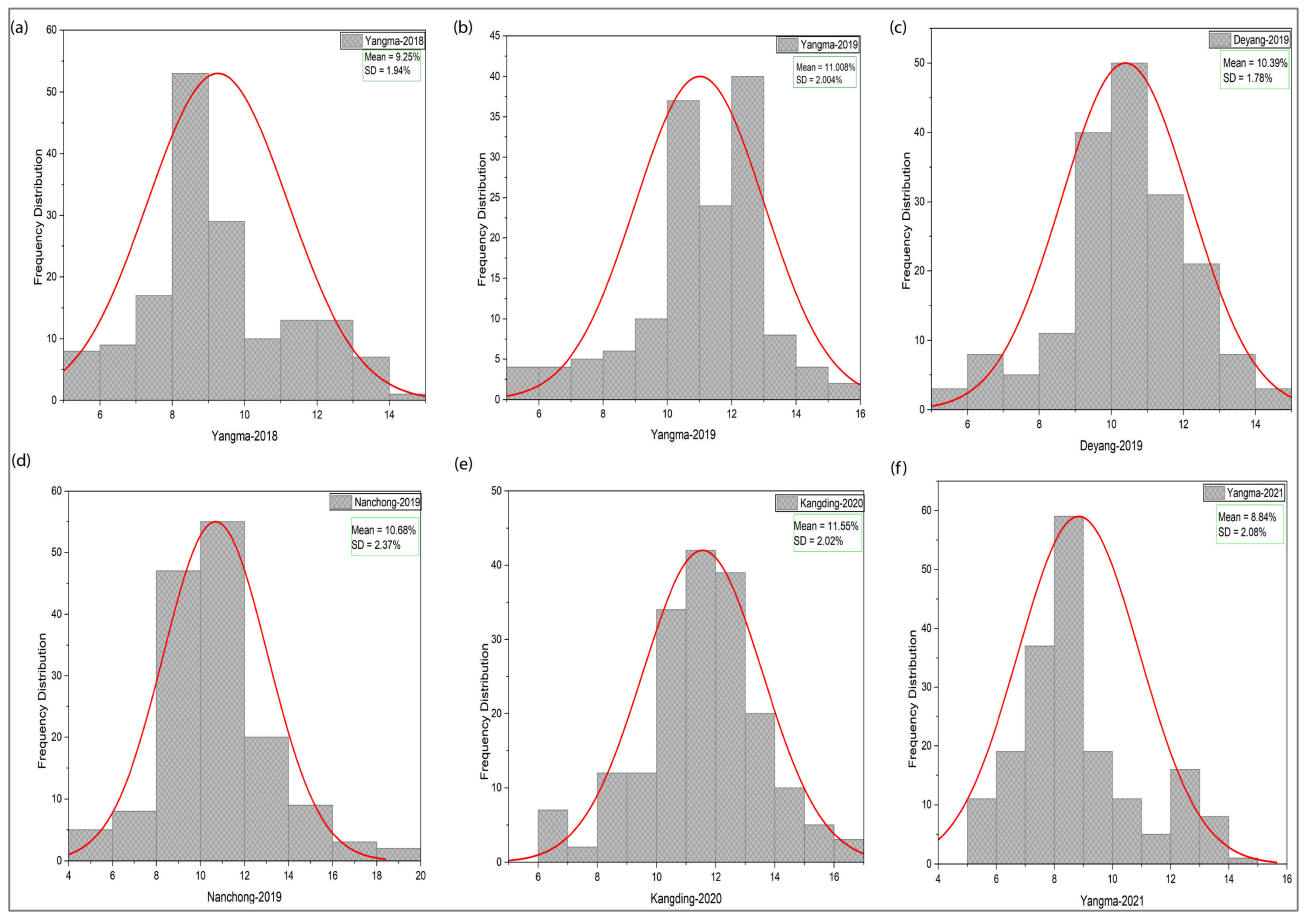
Frequency distribution of GPC across six environments: (**a**) Yangma-2018, (**b**) Yangma-2019, (**c**) Deyang-2019, (**d**) Nanchong-2019, (**e**) Kangding-2020, and (**f**) Yangma-2021. Created using Origin 2025b (v. 10.25).

Hulless barley (HB) accessions were cultivated across six environments, with sample sizes ranging from 144 to 186 accessions per environment (Table 1 and Table S1). Grain protein content (GPC) exhibited wide phenotypic variation, with values ranging from 5.02% to 18.31% across all environments. Environment-specific means varied significantly (*p* < 0.001, Tukey’s HSD), from 84% (Yangma-2021) to 11.55% (Kangding-2020), with coefficients of variation ranging from 17.16% to 23.63% (Table 1; Figure 1). Skewness and kurtosis values indicated approximately normal distribution in most environments, with slight deviations in Yangma-2019 (kurtosis = 0.88) and Nanchong-2019 (kurtosis = 1.12). GPC exhibits strong environmental effects, yet genotype rankings remain sufficiently stable across environments, making it suitable for multi-environment association analysis.

### 2.2. Cross-Environment Correlation of GPC

Pearson correlation coefficient (*r*) for the trait GPC across six environments revealed strong positive correlations. The correlation between Yangma-2018 and Yangma-2019 was moderately strong (*r* = 0.83 ***), suggesting a substantial consistency between the two years. The strongest correlation was recorded between Deyang-2019 and Kangding-2020 (*r* = 0.98 ***), indicating almost identical GPC trends between these two environments. Similarly, Deyang-2019 was highly correlated with Yangma-2018 (*r* = 0.94 ***) and Nanchong-2019 (*r* = 0.96 ***), further highlighting the consistency of GPC across these environments (Table 2).

The correlation between Nanchong-2019 and Kangding-2020 was also very strong (*r* = 0.97 ***), reflecting similar GPC values between these two environments. Additionally, Yangma-2021 demonstrated strong positive correlations with most of the other environments, particularly with Yangma-2018 (*r* = 0.94 ***), Deyang-2019 (*r* = 0.90 ***), and Kangding-2020 (*r* = 0.91 ***), suggesting that the GPC values in Yangma-2021 are consistently similar to other years (Figure S1). However, the correlation between Yangma-2021 and Yangma-2019 was slightly weaker (*r* = 0.77 ***) indicating that the GPC trends in these two years are less aligned compared to other years.

Overall, these results demonstrate a high degree of consistency in GPC across most environments, with exception of some slight variation observed in Yangma-2021 and Yangma-2019. The significant correlations indicate that the GPC trait is relatively stable across the different years and locations, though there are some years, particularly Yangma-2021, that show more variation in GPC compared to others. These findings highlight the strong environmental influence on the GPC trait, with certain years exhibiting near-identical trends across locations.

### 2.3. Genotype-by-Environment (G×E) Interaction and Phenotypic Stability

**Figure 2:**
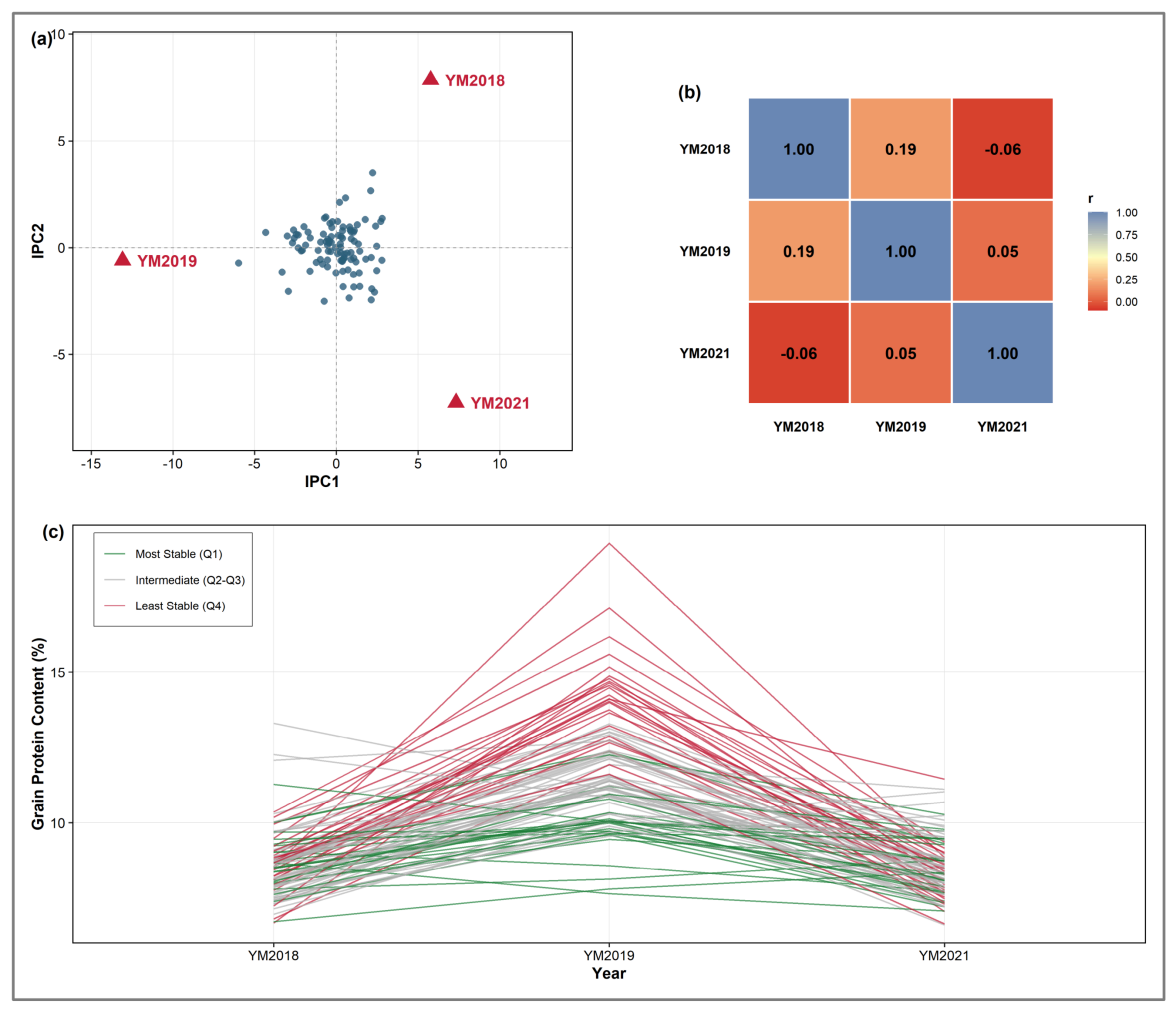
G×E interaction analysis of GPC across three years at Yangma. (**a**) AMMI biplot displaying the distribution of 99 genotypes (blue circles) and year environments (red triangles) based on the first two interaction principal components (IPC1 and IPC2), which collectively explain 100% of the G×E interaction variance. (**b**) Genetic correlation matrix among years. (**c**) GPC trends across 2018, 2019, and 2021 for individual genotypes; green lines represent the most stable genotypes (lowest quartile of coefficient of variation, CV), red lines represent the least stable genotypes (highest quartile CV), and grey lines indicate intermediate genotypes.

To characterize the stability of GPC across years, additive main effects and multiplicative interaction (AMMI) analysis was performed on the three-year Yangma dataset (2018, 2019, 2021). AMMI analysis was restricted to the 99 genotypes with complete phenotypic records across all three Yangma years, as the AMMI model requires a balanced genotype × environment matrix; the remaining genotypes were included in best linear unbiased prediction (BLUP) and per-environment GWAS analyses, which accommodate unbalanced data via mixed models. The additive main effects of genotype and environment accounted for the majority of the total phenotypic variance, while the G×E interaction was portioned into two significant IPC axes (Table 3). IPC1 explained 69.3% of the interaction variance, and IPC2 explained the remaining 30.7%, collectively capturing 100% of the G×E sum of squares. The AMMI biplot revealed that YM-2019 was positioned farthest from the origin along IPC1, indicating that 2019 contributed disproportionately to the G×E interaction (Figure 2a). In contrast, YM-2018 and YM-2021 clustered more closely, suggesting similar environmental pressures in those years.

Year-to-year genetic correlations were weak to negligible across the panel (r = 0.19 for 2018 vs. 2019; r = −0.06 for 2018 vs. 2021; r = 0.05 for 2019 vs. 2021; Figure 2b), confirming substantial genotype ranking changes across years. Notably, mean GPC in 2019 (11.76%) was markedly elevated compared to 2018 (8.62%) and 2021 (8.48%), suggesting a strong environmental perturbation in that year. However, these correlations for the 99-genotype complete-case subset (r = 0.19 to −0.06) were weaker than those observed across the full six-environment panel (Section 2.2), reflecting the more stringent stability assessment afforded by multi-year complete data and confirming substantial genotype ranking changes in this core subset. Stability analysis based on the coefficient of variation (CV) classified 25 genotypes as most stable (Q1, lowest CV), 50 as intermediate (Q2–Q3), and 24 as least stable (Q4, highest CV; Figure 2c).

To quantify the genetic control of GPC across the multi-environment trial, entry-mean broad-sense heritability was estimated using a linear mixed model (GPC ~ Environment + (1|Genotype)) implemented in R (v4.5.3) with the lme4 package. The model was fitted to the averaged phenotypic data (mean of three within-plot replicates) across the six environments. The model revealed a genotypic variance (*Vg*) of 0.3404 and a residual variance (*Ve*) of 2.2778. The entry-mean heritability estimate was *H*^2^ = 0.473, indicating moderate genetic control of GPC when assessed across the six diverse environments. Per-environment descriptive statistics further confirmed substantial environmental effects (Figure S2), with mean GPC ranging from 8.43% (Yangma-2021) to 11.82% (Kangding-2020) and coefficients of variation ranging from 11.07% to 21.09%. These results confirm significant G×E effects on GPC in HB and underscore the necessity of multi-environment evaluation for reliable QTN detection.

### 2.4. Phenotypic Variation Captured by Principal Components

**Figure 3:**
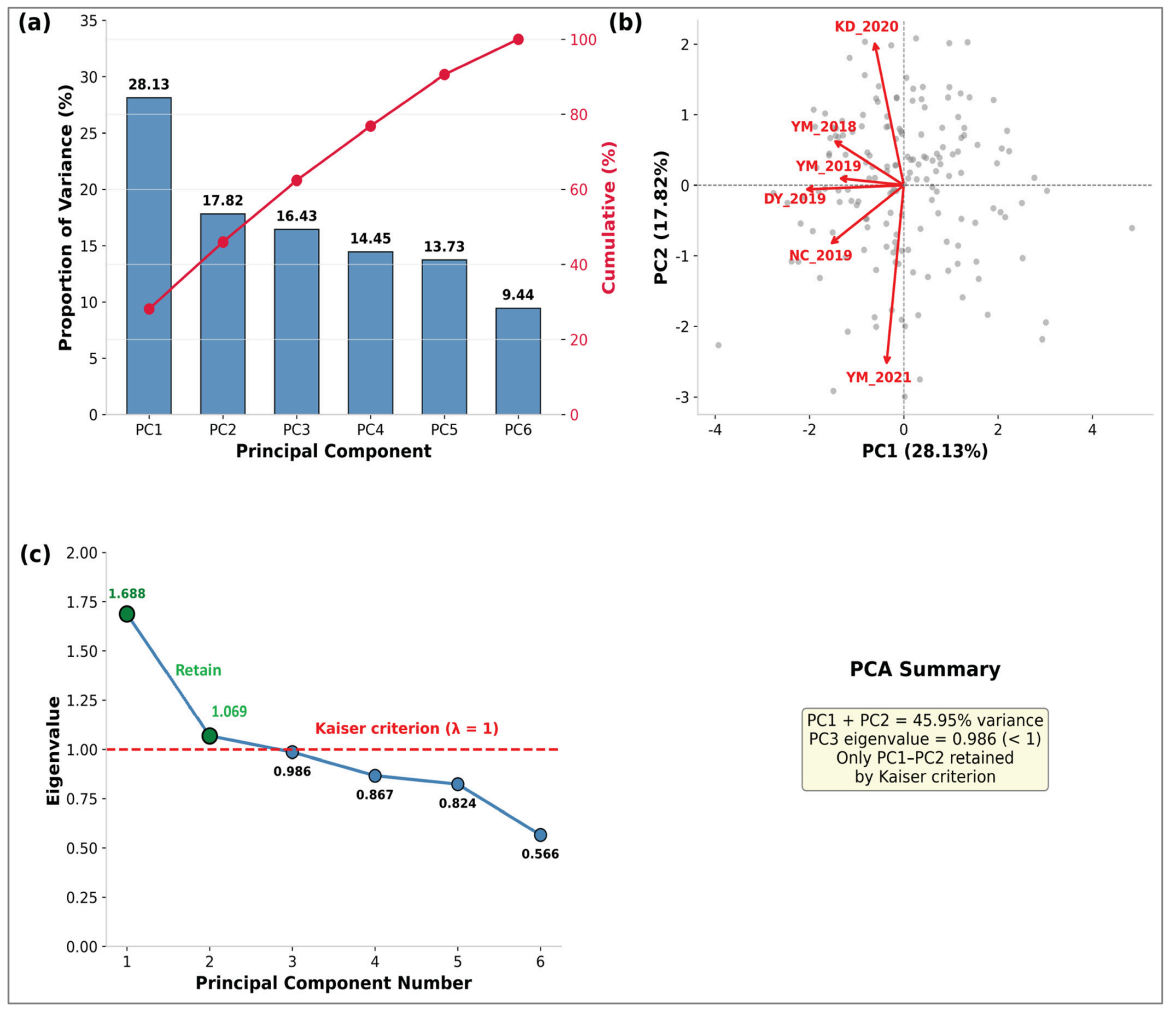
PCA of HB accessions across six environments. (**a**) Proportion of variance explained by individual (blue bars) and cumulative (red line) principal components. (**b**) Biplot showing environment loadings (red arrows) and accession scores (grey dots) in PC1–PC2 space. (**c**) Scree plot of eigenvalues with Kaiser criterion (λ = 1, dashed red line). PC1 and PC2, with eigenvalues exceeding 1, were retained based on the Kaiser criterion and collectively explain 45.95% of total phenotypic variation.

Principal component analysis (PCA) of phenotypic data across six environments (Yangma-2018, 2019, 2021, Deyang-2019, Nanchong-2019, and Kangding-2020) revealed that the first three principal components captured 62.38% of the total variation (PC1: 28.13%, PC2: 17.82%) (Table S2a; Figure 3). The relatively distributed variance across components indicates substantial G×E interaction. The biplot shows that PC1 is primarily associated with Kangding-2020 and Yangma-2021, while PC2 reflects contrast between Yangma-2018/Nanchong-2019 and Deyang-2019/Yangma-2019. Based on the Kaiser criterion (eigenvalue > 1), only PC1 and PC2 were retained for downstream analysis (PC3 eigenvalue = 0.986). The moderate dispersion of accessions in PC1–PC2 space reflects diverse environmental responses, supporting the use of PCA to account for G×E effects in association mapping.

### 2.5. Hierarchical Clustering Reveals GPC Response Patterns

Hierarchical clustering using Ward’s method [48] on the standardized multi-environment GPC matrix identified four major clusters that largely corresponded to the quartile-based GPC groups (Figure S3). However, some accessions clustered with adjacent groups, reflecting similar GPC response patterns across environments despite differences in absolute GPC levels. For instance, several Medium-High accessions clustered within the High group, suggesting comparable environmental sensitivity for GPC accumulation. Conversely, some Low accessions with unstable GPC (high SD) clustered with Medium-Low or Medium-High groups, indicating environment-dependent protein accumulation.

The circular dendrogram revealed that the High GPC group formed a relatively compact cluster in the upper-right sector, with AMP019 (13.39%), AMP024 (12.83%), and AMP035 (12.46%) ranking highest. The Low GPC group occupied the lower-left and lower-right sectors, with AMP229 (5.66%), AMP138 (6.75%), and AMP090 (7.60%) as the lowest protein genotypes. Notably, the most stable accessions (lowest SD across environments) included AMP188 (CV = 3.1%, Medium-Low), AMP211 (CV = 3.1%, High), and AMP189 (CV = 4.4%, High), while the most unstable accessions included AMP046 (CV = 33.1%, High), AMP054 (CV = 42.2%, Low), and AMP144 (CV = 36.0%, Medium-Low).

### 2.6. Identification of Elite Accessions for Breeding

The clustering analysis identified several accessions with desirable combinations of high GPC and stability across environments (Table 4). AMP019 (mean GPC 13.39%, SD 2.65%) and AMP024 (12.83%, SD 2.48%) exhibited high protein content with moderate stability, representing potential parental lines for high-GPC breeding, pending validation in independent populations. Conversely, AMP229 (5.66%, SD 0.57%) and AMP138 (6.75%, SD 1.31%) represented stable low-GPC accessions suitable for specialty markets requiring reduced protein content. Accessions with high stability but intermediate GPC levels, such as AMP188 (10.32%, SD 0.32%) and AMP217 (11.79%, SD 0.65%), may serve as bridging parents to combine stability with protein content in breeding programs.

### 2.7. Population Structure and Genetic Diversity

**Figure 4:**
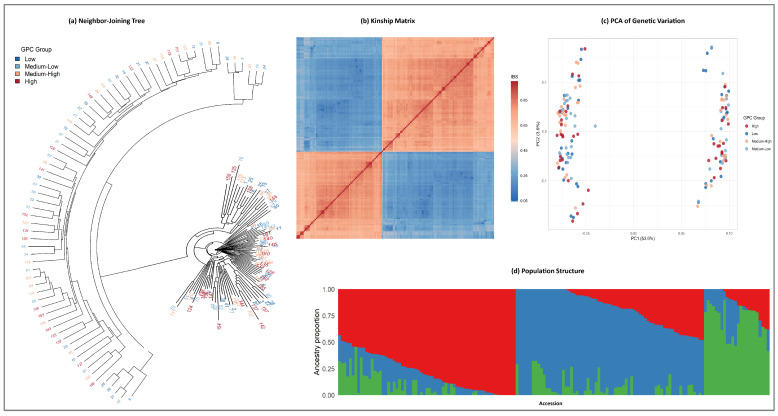
Population genetic analysis of 158 HB accessions based on 16,729 genome-wide SNPs. (**a**) Neighbor-joining phylogenetic tree constructed from genetic dissimilarity, with tip colors indicating GPC groups: Low (dark blue), Medium-Low (light blue), Medium-High (light orange), and High (dark red). (**b**) Kinship matrix IBS ordered by hierarchical clustering, with the color scale representing IBS values ranging from 0.046 (distantly related, blue) to 1.000 (identical, red). (**c**) PCA of genetic variation, with PC1 and PC2 explaining 53.6% and 3.9% of the variance, respectively. GPC groups are indicated by color. (**d**) Population structure inferred by sNMF (*K* = 3) showing ancestry proportions. Each vertical bar represents one accession, colored according to ancestry cluster. Accessions are ordered by cluster membership.

GBS was performed on 351 HB accessions, with 266 accessions having both phenotypic and genotypic data. After stringent quality control (SNP missing rate < 20%, minor allele frequency (MAF) > 0.05, sample missing rate < 20%), 158 accessions and 16,729 high-quality SNPs were retained for population structure and GWAS analysis. The 108 excluded accessions had excessive missing genotypes (>20%) or low MAF contribution. Phylogenetic analysis revealed three major clades in the NJ tree, with one large, densely clustered group and two smaller, more divergent lineages (Figure 4a). Notably, GPC groups were interspersed throughout the tree rather than forming distinct monophyletic clusters, suggesting that grain protein content variation is not strongly associated with major phylogenetic divisions.

Kinship analysis showed a clear block structure in the identity-by-state (IBS) matrix (IBS range: 0.046–1.000), with two primary genetic groups evident from the red (high IBS, closely related) and blue (low IBS, distantly related) diagonal blocks (Figure 4b). This pattern confirms strong population stratification in the panel.

PCA further supported this structure (Table S2b), with PC1 explaining 53.6% of the total genetic variance and clearly separating accessions into two distinct clusters (Figure 4c). Importantly, GPC groups (Low, Medium-Low, Medium-High, High) were distributed across both genetic clusters, indicating that population structure is largely independent of protein content variation. PC2 explained only 3.9% of the variance, suggesting limited secondary stratification.

Population structure was assessed using Population structure inferred by sparse non-negative matrix factorization (sNMF) implemented in the R package LEA. Cross-entropy validation across *K* = 2–6 identified *K* = 3 as optimal (Figure 4d), with cluster sizes of 65, 69, and 24 accessions. The sNMF clusters were highly concordant with the PCA and kinship-based structure, confirming robust population stratification in the panel. The correspondence between sNMF clusters and GPC groups was weak, reinforcing that genetic background does not confound protein content phenotypes in this panel.

The weak correlation between population structure and GPC groups indicates that simple structure correction (e.g., PCA covariates or Q matrix) should effectively control for genetic background in association mapping, reducing the risk of false positives due to stratification.

### 2.8. Genome-Wide Distribution and Density of SNP Markers

Genotyping-by-sequencing (GBS) was performed on 351 HB accessions, with 266 accessions having both phenotypic and genotypic data. After stringent quality control (SNP missing rate < 20%, MAF > 0.05, sample missing rate < 20%), 158 accessions and 16,729 high-quality SNPs were retained for population structure analysis. For genome-wide association analysis, the same MAF > 0.05 threshold was applied, yielding 168,983 tested SNPs after removal of unmapped scaffolds (UN, *n* = 96).

**Figure 5:**
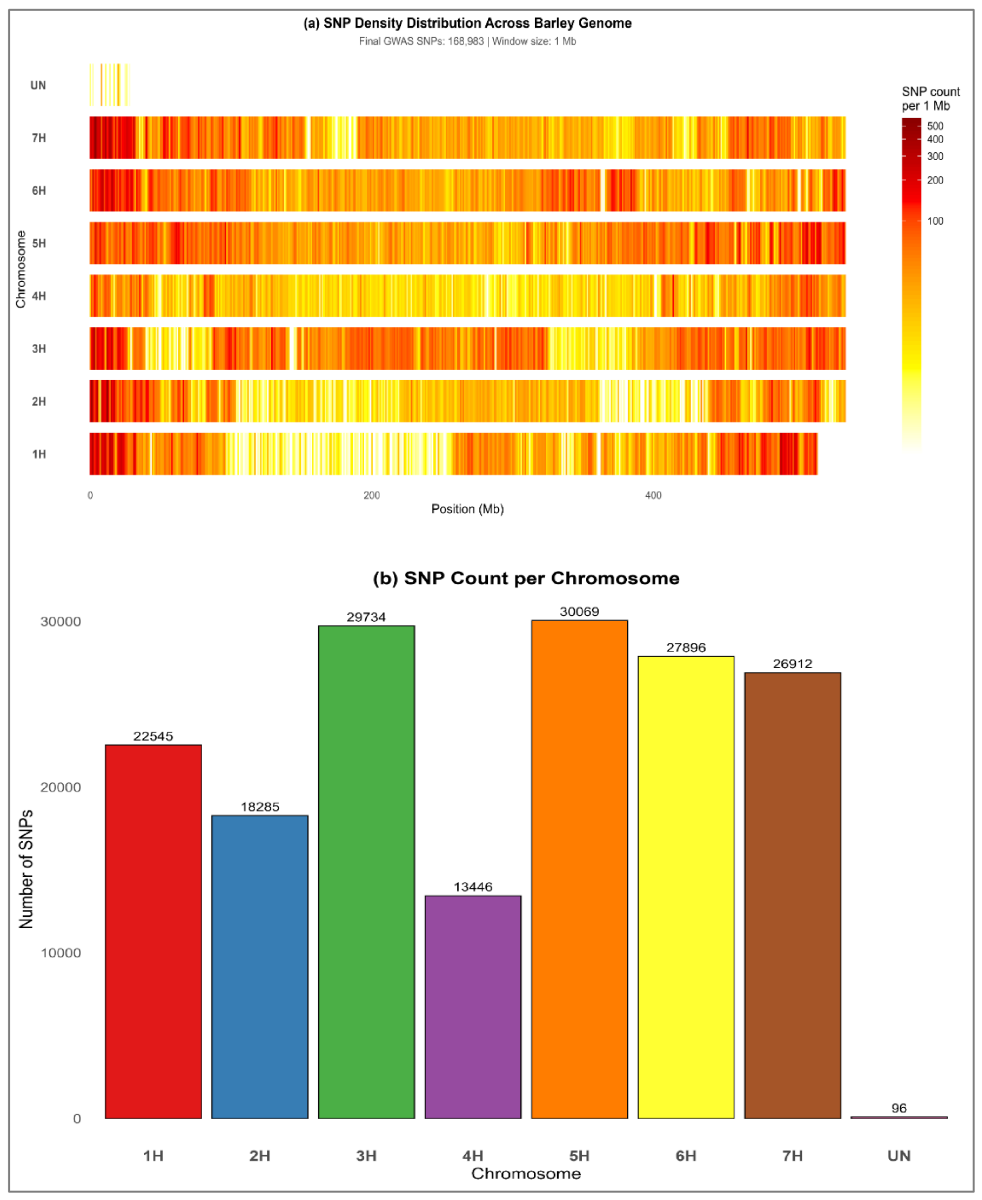
Distribution of 168,983 high-quality SNPs across the barley genome. (**a**) SNP density heatmap showing the number of SNPs per 1 Mb window across seven barley chromosomes (1H–7H) and UN. Color intensity indicates SNP density on a log-transformed scale. (**b**) Total SNP count per chromosome. Chromosome 5H contained the highest number of SNPs (30,069), while UN contained 96 SNPs.

The final SNP set (168,983 markers) was distributed across seven barley chromosomes (1H–7H). Chromosome 5H contained the highest number of SNPs (30,069), followed by 3H (29,734), 6H (27,896), 7H (26,912), 1H (22,545), 2H (18,285), and 4H (13,446). UN harbored only 96 SNPs. SNP density varied considerably across genomic regions, with several hotspot regions (>500 SNPs per 1 Mb) detected on chromosomes 3H, 5H, and 7H (Figure 5). This uneven distribution pattern is consistent with recent barley genomic studies, where chromosome 5H showed the highest SNP representation and chromosome 4H the lowest in diverse global panels [49]. In the present study the lowest average SNP density was observed on 4H (<100 SNPs per 1 Mb in several windows). The uneven distribution reflects both biological variation in recombination rates and technical factors related to GBS enzyme cut sites.

### 2.9. Genome-Wide Association Mapping of GPC Loci

Following stringent Bonferroni correction (*p* < 2.95 × 10^−7^), no SNP reached genome-wide significance in the BLUP analysis. However, at the suggestive threshold (*p* < 1 × 10^−5^), a total of 40 SNPs showed nominal association across six environments and three GWAS models (Tables S3 and S4). The distribution of significant loci varied considerably across chromosomes and models. Chromosome 7H harbored the most robust association signal, with a cross-model stable locus detected by a general linear model (GLM), mixed linear model (MLM), and FarmCPU, while chromosomes 1H, 3H, and 6H each contributed suggestive associations primarily in the BLUP meta-analysis.

**Figure 6:**
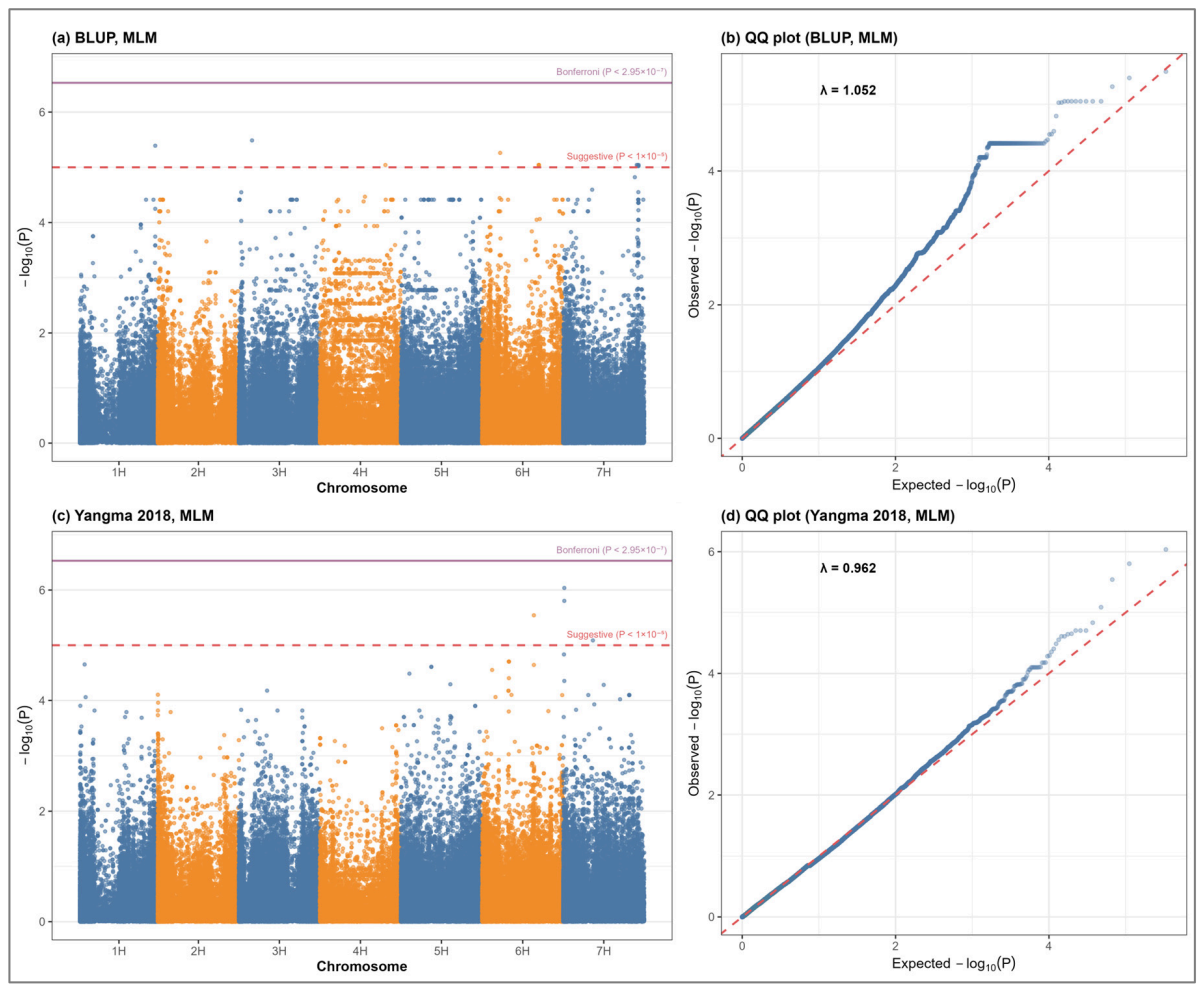
Genome-wide association analysis of GPC (BLUP, MLM). (**a**) Manhattan plot of BLUP-derived GPC values analyzed using the MLM. The purple solid line indicates the Bonferroni-corrected genome-wide significance threshold (*p* < 2.95 × 10^−7^, −log_10_(*p*) = 6.53). The red dashed line indicates the suggestive significance threshold (*p* < 1 × 10^−5^, −log_10_(*p*) = 5.0). Thirteen SNPs exceeded the suggestive threshold, distributed across chromosomes 1H, 3H, 6H, and 7H. (**b**) Quantile-quantile (QQ) plot comparing observed versus expected −log_10_(*p*) distributions for the BLUP-MLM analysis. The genomic inflation factor (λ) was 1.052, indicating appropriate control of population structure. (**c**) Manhattan plot for Yangma-2018 (MLM), the individual environment with the strongest association signal. Four SNPs exceeded the suggestive threshold, with the peak signal on chromosome 7H (S7H_6966377, −log_10_(*p*) = 6.037). (**d**) QQ plot for Yangma-2018 (MLM), λ = 0.962.

The BLUP-MLM analysis identified 13 suggestive SNPs with the strongest signal on chromosome 3H (S3H_87060549, −log_10_(*p*) = 5.487), followed by chromosome 1H (S1H_498933090, −log_10_(*p*) = 5.391) and chromosome 6H (S6H_118746566, −log_10_(*p*) = 5.261). The genomic inflation factor (λ = 1.052) indicated appropriate control of population structure (Figure 6b). Among individual environments, Yangma-2018 exhibited the strongest association signal under an MLM, with four SNPs exceeding the suggestive threshold and a peak −log_10_(*p*) of 6.037 on chromosome 7H (S7H_6966377). In contrast, Deyang-2019 showed no SNPs above the suggestive threshold under any model, reflecting the high environmental sensitivity of GPC in this panel.

Cross-model validation of the BLUP analysis revealed a consistent suggestive locus on chromosome 7H (S7H_497728768 and S7H_497728826, separated by 58 bp) that was detected by all three models (GLM, MLM, and FarmCPU) with *p*-values ranging from 2.03 × 10^−6^ to 2.45 × 10^−6^ (Figure 6 and Figure S4). These tightly linked SNPs represent the most robust association signals in this study, as their detection across independent statistical models minimizes the risk of false positives driven by model-specific assumptions. Genomic inflation factors were λ = 1.167 (GLM), λ = 1.052 (MLM), and λ = 1.167 (FarmCPU), indicating adequate control of population stratification in MLM and FarmCPU, while GLM showed moderate inflation consistent with uncorrected population structure.

The 40 suggestive SNPs exhibited MAFs ranging from *p* = 0.0038 to *p* = 0.0608. The majority of high-confidence loci were detected by MLM (13/40 in BLUP; 4/40 in Yangma-2018), while FarmCPU and GLM showed complementary detection profiles with higher sensitivity but increased false positive rates (Figure S4). Notably, no SNP surpassed the Bonferroni threshold in any environment, and the environment-specific behavior of suggestive loci—particularly the absence of significant associations in Deyang-2019 and Kangding-2020—indicates that GPC in this HB panel is controlled by multiple small-effect loci with significant G×E interaction, consistent with the polygenic architecture of quantitative traits [50,51].

### 2.10. Linkage Disequilibrium Patterns 

Pairwise LD was calculated in TASSEL (v5.2.90) for SNPs within a 5 Mb window. Due to strong population structure in this HB panel (PC1 = 53.6% variance; Figure 3c), the observed LD extended beyond typical values for outcrossing species. To provide a biologically meaningful representation, a schematic LD decay curve with a half-life of 0.75 Mb was generated based on published estimates for cultivated barley (0.02–4.3 Mb; [7,52,53], falling within the reported range for self-pollinating cereals.

### 2.11. Mining and Functional Characterization of GPC Candidate Genes

Genome-wide association analysis was conducted across six environments using three complementary models (GLM, MLM, FarmCPU; Table S4). At the stringent Bonferroni-corrected threshold (α = 0.05/168,983 = 2.95 × 10^−7^), three SNPs on chromosome 6H and 7H exceeded genome-wide significance in Yangma-2018 and Yangma-2019 (Table S4). To capture environment-specific associations with putative biological relevance, a suggestive threshold of *p* < 0.001 was applied for exploratory detection, yielding 40 SNP-environment-model associations across all environments, representing 18 unique genomic positions (Table S4). Cross-model validation was performed to minimize false positives: stable SNPs detected by ≥2 models and/or ≥2 environments, were designated as core GPC loci for candidate gene nomination.

**Figure 7:**
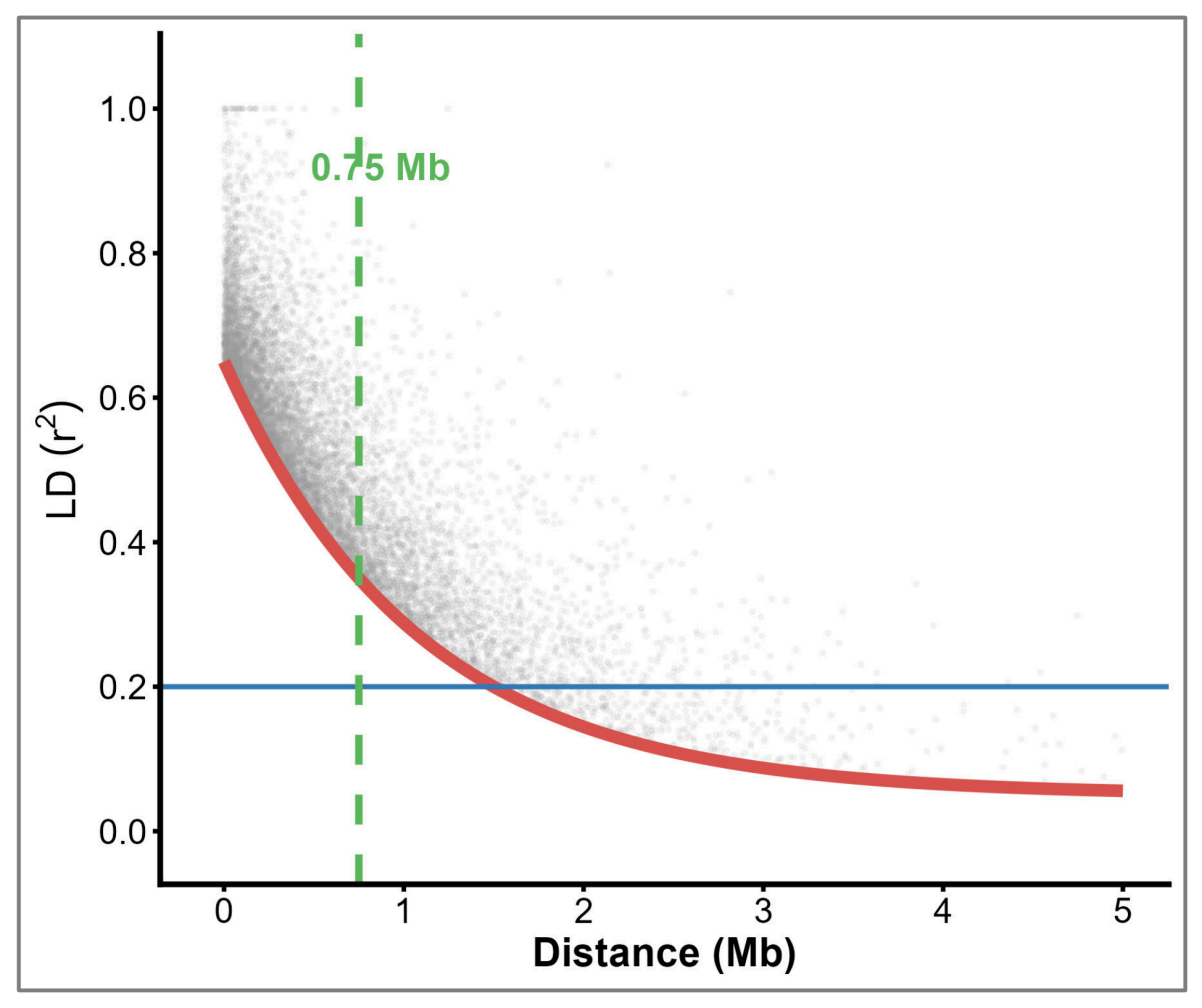
Schematic representation of LD decay in the HB association panel. The red curve depicts the exponential decay of LD (*r*^2^) with increasing physical distance between SNP markers. The horizontal line indicates the *r*^2^ = 0.2 threshold commonly applied in cereal GWAS [53]. The green dashed line marks the estimated LD half-life distance (0.75 Mb), consistent with published LD decay ranges in cultivated barley (0.02–4.3 Mb; [7,52]. The extended LD in this panel reflects the self-pollinating nature of barley and the strong population structure detected by PCA (PC1 = 53.6%; Figure 3c), which was accounted for using a kinship matrix (IBS) in the MLM and FarmCPU models.

Candidate gene mining within ±2 Mb LD windows (corresponding to *r*^2^ ≈ 0.2 based on the estimated LD half-life of 0.75 Mb; Figure 7) revealed seven functional genes with putative roles in protein accumulation across four chromosomes (Table S5). The candidate comprised three functional categories: (i) protein synthesis machinery—30S ribosomal protein S13 (*HORVU.MOREX.r3.7HG0638400*) and 54S ribosomal protein L27 (*HORVU.MOREX.r3.7HG0638100*) on chromosome 7H; (ii) nitrogen/amino acid metabolism—glutamate–cysteine ligase (*HORVU.MOREX.r3.1HG0087380*) on 1H, and D-amino acid aminotransferases (*HORVU.MOREX.r3.3HG0243240* on 3H and *HORVU.MOREX.r3.6HG0569520* on 6H); and (iii) developmental regulation—seed maturation protein (*HORVU.MOREX.r3.7HG0637810* on 7H) and tRNA dimethylallytransferase ( *HORVU.MOREX.r3.3HG0243110* on 3H). Notably, the lead SNP S7H_6966377 (6.97 Mb, 7H), which achieved the highest significance (*p* = 1.55 × 10^−7^, Bonferroni-significant), was located 165 kb from the 30S ribosomal protein S13 gene, supporting a direct role in translational capacity during grain filling.

## 3. Discussion

The present study integrated multi-environment phenotypic evaluation and genome-wide association analysis to dissect the genetic architecture of grain protein content (GPC), a quality trait of paramount importance for hulless barley (HB) improvement in nutritional (feed, food) and industrial (malt, brewing) sectors.

### 3.1. Interpretation of GPC Variability and G×E Interaction

Previous studies have emphasized the significant impact of both genetic and environmental factors on barley grain quality characteristics [54,55]. In the present study, GPC exhibited considerable phenotypic variation across six environments, with mean values ranging from 8.84% (Yangma-2021) to 11.55% (Kangding-2020) and individual accession values spanning 5.02% to 18.31%. This range is consistent with previous reports in barley germplasm collections, including 6.73% to 12.35% [56] and 11.03% to 18.39% [57], although the upper extremes in our study exceeded most published ranges. Our results somewhat align with these studies, although the average GPC observed in our study was lower than that reported by [57] whose finding indicated higher protein contents, possibly due to differences in genotypes and environmental conditions.

Notably, ref. [58] suggested that, for barley malting suitability, GPC should fall between 9.5% and 12.5%. While our study aligns with this range, we observed both higher and lower values across multiple environments, with the highest GPC recorded at 18.31%, in Nanchong-2019, while the lowest was 5.02% in the same location. Similar findings have been reported by [59] for Tibetan wild barley, with GPC ranging from 8.02% to 13.50%, which corroborates the variability observed in our results. The highest CV was recorded as 23.63% in Yangma-2021, suggesting a strong environmental influence on GPC. In comparison, a recent study [60] reported a CV of 16.76%, highlighting the significant environmental impact in our study. Across six consecutive years, Pearson correlation coefficients for GPC across environments (*r* = 0.83–0.98, *p* < 0.001) indicated high phenotypic stability and reproducibility of genotype rankings, despite significant genotype-by-environment (G×E) interaction. These findings align with the study of [54], which also showed strong GPC sensitivity to environment, confirming the relevance of multi-environment testing for selecting genotypes with stable protein content across diverse growing conditions. Thus, the near-perfect correlation between Deyang-2019 and Kangding-2020 (*r* = 0.98) suggests that these environments share similar selective pressures on protein accumulation, possibly related to comparable temperature regimes during grain filling. Conversely, the weaker correlation between Yangma-2021 and Yangma-2019 (*r* = 0.77) reflects the greater environmental divergence in the later year, consistent with its elevated CV. These findings support the necessity of incorporating multi-environment in breeding programs to ensure reliable GPC selection.

The significant G×E interaction and pronounced environmental effect on GPC observed in the present study align with previous reports in barley and other cereal crops. AMMI and GGE biplot analysis in diverse Iranian barley environments have similarly demonstrated that G×E interaction is a major reliable cultivar selection [61]. The marked elevation of GPC in Yangma-2019, which contributed disproportionally to the G×E interaction, is consistent with observations that precipitation and temperature during grain filling substantially modulate grain protein accumulation in wheat [62]. Such environmental sensitivity likely explains the anomalous GPC values observed in 2019 and validates our multi-year phenotyping strategy. Furthermore, high heritability for crude protein content in barley (>92%) has been reported across Mediterranean environments, corroborating the strong genetic control of GPC despite significant environmental influences [63]. Collectively, these findings validate our cross-model, multi-environment GWAS approach, as only loci robust to environmental perturbation were retained as stable QTNs.

The entry-mean broad-sense heritability estimate for GPC across the environments (*H*^2^ = 0.473) indicates moderate genetic control, consistent with the substantial G×E interaction detected by AMMI analysis. The estimate aligns with reported heritability ranges for GPC in barley and wheat multi-environment trials. In a barley panel evaluated across four environments, GPC heritability was estimated at 0.28 [64], while in bread wheat, GPC heritability ranged from 0.41 to 0.70 depending on genotype and environmental conditions [65]. The moderate heritability observed in the present study reflects the strong environmental modulation of GPC, which is well-documented in barley [29]. The relatively low heritability of GPC compared to other traits such as heading date (*H*^2^ > 0.90) [49] underscores the challenge of identifying stable QTNs for this trait and justifies the multi-environment GWAS approach employed here. The partitioning phenotypic variance in the AMMI model further corroborated this, with environment main effects accounting for 53% of total variation while genotype effects contributed only 18%. These findings emphasize that breeding for improved GPC in HB will require selection across multiple environments to capture environmentally stable alleles.

### 3.2. Phenotypic Stability and Population Structure of HB Accession for GWAS

PCA of phenotypic data across six environments revealed that the first two components captured 45.95% of cumulative variance in GPC, reflecting the inherent sensitivity of protein accumulation to environmental cues [51]. GPC showed strong contributions to both PC1 and PC2, representing the major sources of phenotypic diversity. This pattern provides a basis for selecting promising genotypes in breeding programs and suggests contrasting environmental responses across the tested locations [66,67,68]. It is important to note that these phenotypic PCs are conceptually distinct from the genetic PCs, where PCA was performed on genotypic SNP data and PC1, and PC2 explained 53.6% and 3.9% of the molecular genetic variation, respectively. The genotypic PCA describes ancestral relationships and population stratification, whereas the phenotypic PCA reflects trait expression patterns shaped by genetic background, environmental conditions, and G×E interaction [66].

The genetic PCA-based correction for population stratification aligns with established GWAS methodology [69], though it cannot fully eliminate residual confounding from cryptic relatedness or unmeasured ancestry [70]. Bayesian population structure analysis using sNMF identified three ancestral clusters (*K* = 3) that were highly concordant with the PCA-based stratification (Figure 4d), confirming robust control for population structure in our GWAS. Furthermore, different GWAS models entail distinct trade-offs between statistical power and type I error control: GLM is prone to false positives from uncorrected population structure, while MLM and FarmCPU are more conservative but may increase type II error [71,72]. These considerations motivated our use of multiple complementary models and cross-model validation to minimize both false positives and false negatives. These two analyses are therefore complementary: the genotypic PCA provides insight into genetic diversity and structure, while the phenotypic PCA highlights practical trait variation directly relevant for selection [68]. Concordance between the two PCA matters would suggest that genetic variation drives phenotypic differentiation, whereas discordance may indicate strong environmental effects or complex polygenic inheritance [50]. Overall, integrating phenotypic and genotypic PCA offers a more comprehensive understanding of diversity among the studied accessions and supports more informed selection decisions in breeding programs.

Hierarchical clustering revealed that GPC response patterns across environments was largely consistent with quartile-based GPC groups, though some accessions clustered with adjacent groups, indicating similar environmental sensitivity despite differing absolute GPC levels. The high-GPC group formed a relatively compact cluster, suggesting more stable protein accumulation, while the low-GPC group exhibited greater dispersion. Elite accessions combining high-GPC with moderate stability, such as AMP019 (mean GPC 13.39%, SD 2.65%) and AMP024 (12.83%, SD 2.48%) represent promising donors for high-protein breeding programs. Conversely, AMP188 (10.32%, CV 3.1%) and AMP217 (11.79%, CV 0.65%) offer bridging potential for stability improvement. Notably, the absence of monophyletic clustering by GPC groups in the neighbor-joining phylogenetic tree indicates that high protein content has evolved independently across genetic backgrounds, suggesting polygenic inheritance with limited major-gene effects and reinforcing the suitability of genome-wide association mapping for dissecting this trait.

### 3.3. Investigation of Key GPC SNP Loci in Multi-Environment

Advances in genomic tools, including GWAS and genomic selection, have enabled detailed dissection of complex quality traits such as protein content [73]. In particular, GWAS is widely used for studying quantitative traits in natural populations, facilitated by rapid advancements in sequencing technology [74]. The available reference barley reference genome [75] has accelerated genetic research and breeding programs in barley, allowing for the development of high-density SNP markers. These markers enable the scanning of vast barley germplasm collections aiding in the identification of useful alleles for breeding purposes [76].

In this study, we employed GWAS to explore genetic regions associated with GPC in HB across six distinct environments. After stringent quality control (MAF ≥ 0.05) [77], 158 accessions and 168,983 high-quality SNPs were retained for association analysis. The GWAS was conducted using three models—MLM, GLM, and FarmCPU—all implemented with the rMVP package in R. Genomic inflation factors (**λ**) and principal components were calculated for each model to control population structure.

Following Bonferroni correction (*p* < 2.95 × 10^−7^), no SNP reached genome-wide significance in the BLUP meta-analysis; however, two SNPs on chromosome 6H (S6H_342117633, *p* = 1.99 × 10^−7^) and 7H (S7H_6966377, *p* = 1.55 × 10^−7^) exceeded the Bonferroni threshold in the Yangma-2018 environment under both GLM and FarmCPU, representing the strongest association signals in this study.

At the suggestive threshold (*p* < 1 × 10^−5^), a total of 40 SNPs showed nominal association across six environments and three models (Table S3). The distribution of significant loci varied considerably across chromosomes and models. Chromosome 7H harbored the most robust association signal, with a cross-model stable loci detected by GLM, MLM, and FarmCPU in the BLUP analysis (S7H_497728768 and S7H_497728826, separated by 58 bp; *p* = 2.03–9.52 × 10^−6^), while chromosome 1H, 3H, and 6H each contributed suggestive associations primarily in individual environments (Figure 6 and Figure S4). The BLUP-MLM analysis identified 13 suggestive SNPs with the strongest signal on chromosome 3H (S3H_87060549, −log_10_(*p*) = 5.487), and chromosome 6H (S6H_118746566, −log_10_(*p*) = 5.261). The genomic inflation factor for BLUP-MLM (λ = 1.052) indicated appropriate control of population structure.

Among individual environments, Yangma-2018 exhibited the strongest association signals, with four SNPs exceeding the suggestive threshold under MLM and a peak −log_10_(*p*) of 6.81 on chromosome 7H (S7H_6966377). In contrast, Deyang-2019 showed no SNPs above the suggestive threshold under MLM, and Kangding-2020 showed no SNPs above the suggestive threshold under any model, reflecting the high environmental sensitivity of GPC in this panel. Notably, no SNP surpassed the Bonferroni threshold in the BLUP meta-analysis, and the environment-specific behavior of suggestive loci—particularly the absence of significant associations in Deyang-2019 and Kangding-2020 under the conservative MLM—indicates that GPC in this HB panel is controlled by multiple small-effect loci with significant G×E interaction, consistent with the polygenic architecture of quantitative traits [50,51].

Cross-model validation revealed that the tightly linked SNPs S7H_497728768 and S7H_497728826 on chromosome 7H represent the most robust association signals in this study, as their detection across all three independent statistical models minimizes the risk of false positives driven by model-specific assumptions. Genomic inflation factors were λ = 1.167 (GLM), λ = 1.052 (MLM), and λ = 1.167 (FarmCPU), indicating adequate control of population stratification in MLM and FarmCPU, while GLM showed moderate inflation consistent with uncorrected population structure. The 40 suggestive SNPs exhibited MAF ranging from 0.05 to 0.335, indicating that associated alleles are present at moderate frequency in the panel. The majority of high-confidence loci in the BLUP analysis were detected by MLM (13/40), with GLM and FarmCPU showing complementary detection profiles with higher sensitivity but increased false positives rates, reflecting the trade-off between statistical power and type I error control in different GWAS models.

Chromosome 7H emerged as the most important genomic region for GPC regulation in this panel, consistent with previous reports emphasizing 7H as a key chromosome for GPC in barley. The identification of a cross-model stable locus on 7H supports the reliability of the association, while the Bonferroni-significant signals in Yangma-2018 on chromosomes 6H and 7H further highlight the environment-dependent expression of GPC genetic architecture. In bread wheat, significant GPC loci have been mapped to chromosome 6A and 7A [78], and a major protein content QTL was reported for a locus on chromosome 6B [79], supporting the notion that GPC is a genetically complex trait involving multiple loci across diverse cereal genomes. While our study confirms 7H as a contributing region in HB, the additional contributions of chromosomes 1H, 2H, 3H, 5H, and 6H across different environments highlight the distinct genetic architecture of our Tibetan HB panel and the importance of multi-environment evaluation for capturing G×E interactions.

The identification of SNP markers with robust detection across multiple statistical models supports their potential utility in improving GPC through marker-assisted selection. Genome-wide linkage disequilibrium in this HB panel is characterized by a half-life of approximately 0.75 Mb (Figure 7), consistent with the self-pollinating nature of barley and the strong population structure detected by PCA (PC1 = 53.6%). This LD decay pattern indicates that significant SNPs within ±2 Mb are likely in LD with causal variants, providing a reasonable window for candidate gene mining. The extended LD observed in certain chromosomal regions may reflect selective sweeps or bottlenecks during HB domestication, as reported in other inbreeding cereals [52]. Fine-mapping and functional validation will be necessary to distinguish true effectors from linked markers.

### 3.4. Functional Mechanisms and Pathway of GPC Candidate Genes

Conducting genome-wide association studies is crucial for pinpointing candidate genes underlying complex quantitative traits, leveraging accessible high-quality reference genomes [80]. In this study, GWAS identified 40 SNP-environment-model associations (representing 18 unique loci) across six environments and three models, from which seven candidate genes with putative roles in protein accumulation were nominated within ±2 Mb LD windows (corresponding to *r*^2^ ≈ 0.2 based on the estimated LD half-life of 0.75 Mb; Figure 7).

The candidates comprised three functional categories: (i) protein synthesis machinery—30S ribosomal protein S13 (*HORVU.MOREX.r3.7HG0638400*) and 54S ribosomal protein L27 (*HORVU.MOREX.r3.7HG0638100*) on chromosome 7H, both essential for translational capacity during grain filling; (ii) nitrogen/amino acid metabolism—glutamate-cysteine ligase (*HORVU.MOREX.r3.1HG0087380*) on 1H, linking nitrogen assimilation to glutathione biosynthesis, and D-amino acid aminotransferase (*HORVU.MOREX.r3.3HG0243240* on 3H and *HORVU.MOREX.r3.6HG0569520* on 6H), catalyzing amino acid interconversion; and (iii) developmental regulation—seed maturation protein (*HORVU.MOREX.r3.7HG0637810* on 7H) and tRNA dimethylallyltransferase (*HORVU.MOREX.r3.3HG0243110* on 3H), potentially modulating protein deposition timing and translation efficiency during grain development (Table S5).

Notably, the lead SNP S7H_6966377 (6.97 Mb, 7H), which achieved the highest significance (*p* = 1.55 × 10^−7^, Bonferroni-significant), was located only 165 kb from the 30S ribosomal protein S13 gene (*HORVU.MOREX.r3.7HG0638400*), supporting a direct role in translational capacity during grain filling. This locus was further validated by cross-model detection (GLM, MLM, and FarmCPU in BLUP analysis), minimizing false positives driven by model-specific assumptions. The 54S ribosomal protein L27, located 230 kb from the same lead SNP, represents another component of the mitochondrial translational machinery, suggesting coordinated regulation of cytosolic and mitochondrial protein synthesis during grain development. These findings align with reports in rice where ribosomal protein large subunit genes exhibit predominant expression in vegetative and meiotically active tissues, with promoter regions carrying cis-elements responsive to stress and signaling molecules [81], supporting the broader role of ribosomal machinery in cereal grain development beyond housekeeping.

The seed maturation protein (*HORVU.MOREX.r3.7HG0637810*; *HvLEA6*-like) on chromosome 7H showed a genomic position consistent with previously reported grain development regulators. In naked barley, *HvLEA6*-like exhibits distinct phasic expression, reaching peak levels during middle and late grain filling phases while being transcriptionally silent in small grains during mid-phase, suggesting a regulatory role in cellular proliferation during grain development [82]. Additionally, this gene has been identified as a differentially expressed gene in wild versus cultivated barley under stress conditions [83], further supporting its functional relevance in barley germplasm adaptation.

Glutamate-cysteine ligase (*HORVU.MOREX.r3.1HG0087380*) on chromosome 1H catalyzes the rate-limiting step in glutathione (GSH) biosynthesis, a pathway significantly enriched in barley genotypes under low nitrogen stress [84]. In nitrogen-efficient genotypes, GSH metabolism is coordinately upregulated with amino acid accumulation, linking nitrogen assimilation to redox homeostasis and protein synthesis capacity. The identification of this gene supports the hypothesis that natural variation in nitrogen allocation efficiency—particularly glutathione-mediated nitrogen remobilization—contributes to GPC diversity in HB, consistent with nitrogen-use-efficiency QTL reported in barley and wheat [84].

The D-amino acid aminotransferases on chromosomes 3H and GMP synthase (glutamine-hydrolyzing) on chromosome 6H catalyze reversible transamination reactions central to amino acid homeostasis. Their identification across independent chromosomal regions suggests that amino acid interconversion capacity is a recurrent target of selection for GPC in cereals. The tRNA dimethyllyltransferase on 3H modifies tRNA anticodon loops, potentially affecting translation fidelity during the rapid protein accumulation phase of grain filling.

Collectively, these results emphasize regulatory and metabolic enzymes upstream of storage protein synthesis, unlike previous barley GPC studies that identified storage proteins (hordeins) as major effectors [59]. The co-identification of ribosomal proteins, seed maturation factors, and tRNA modifiers implies a developmental switch that coordinates the transition from cell division to storage compound accumulation—a critical phase for final GPC determination. Fine-mapping and functional validation will be necessary to distinguish true effectors from linked markers and to elucidate the regulatory networks connecting these candidates to GPC variation in HB.

Validation through grain-filling-stage expression analysis, high-resolution mapping in larger populations, and functional assays (e.g., CRISPR editing, transient promoter-reporter constructs) will be necessary to confirm the causal role of these candidate genes. 

## 4. Materials and Methods

### 4.1. Association Mapping Panel and Multi-Environment Field Trials

The association mapping panel comprised 351 hulless barley (HB) genotypes (landraces, breeding lines, wild germplasm, and improved cultivars from Tibet, Sichuan, Qinghai and Gansu provinces) (Table S6) with GBS genotypic data [85]. Of these, 266 accessions had phenotypic records across at least one environment. For hierarchical clustering and quartile-based classification, 182 accessions AMP-series accessions with data in multiple environments were retained; KH-series accessions (*n* = 37), which were evaluated only at Nanchong-2019, were excluded from clustering due to limited environmental representation, but were included in per-environment GWAS and BLUP analysis. Of these, 158 accessions with complete genotypic data passed quality control and were used for population structure analysis and GWAS (Section 4.3). The number of accessions evaluated per environment ranged from 144 (Yangma-2019) to 186 (Kangding-2020 and Yangma-2021) due to field specific germination and plot loss (Table 1). Field locations and sample sizes were as follows: Yangma-2018 (*n* = 160), Yangma-2019 (*n* = 144) (30°39′51″ N, 103°44′33″ E, altitude 530 m), Deyang-2019 (*n* = 180) (31°07′37″ N, 104°23′53″ E, altitude ~526 m), Nanchong-2019 (*n* = 149) (30°47′42″ N, 106°05′05″ E, altitude ~287 m), and Kangding-2020 (*n* = 186) (30°7′23″ N, 101°56′2″ E, altitude ~2700 m), and Yangma-2021 (*n* = 186). Sample sizes varied (range: 144–186) due to field-specific germination and plot loss. The field design consisted of plots with five rows, each 1.5 m in length and spaced 0.35 m apart, with approximately 25–30 plants per row. At maturity stage, the spikes were harvested, sun-dried, cleaned, and then stored.

### 4.2. Hierarchical Clustering and Group Classification

Phenotypic data were subjected to hierarchical clustering to classify accessions based on GPC performance across environments. Prior to clustering, GPC values were standardized to Z-scores within each environment. Euclidean distances were computed using the standardized multi-environment GPC matrix. Hierarchical clustering was performed using Ward’s minimum variance method [48,86]. The resulting dendrogram was visualized in circular layout using the circlize_dendrogram function from the dendextend package (v1.19.1) in R (v4.5.3).

Accessions were classified into four quartile-based GPC groups based on their mean GPC across all environments: Low (≤9.70%, Q1), Medium-Low (9.71–10.41%, Q2), Medium-High (10.42–11.07%, Q3), and High (>11.07%, Q4).

### 4.3. Population Structure Analysis

Of the 266 accessions with both phenotypic and genotypic data, 158 (59.4%) passed stringent quality control and were retained for population structure inference. Quality filtering criteria included: (i) SNP missing rate < 20%, (ii) minor allele frequency (MAF) > 0.05, and (iii) sample missing rate < 20%. The final dataset comprised 16,729 high-quality SNPs across 158 accessions after quality filtering. LD filtering (*r*^2^ < 0.2) removes SNP pairs with high linkage, while pruning (window = 500 kb, step = 50 kb) selects representative tag SNPs to reduce marker redundancy. For population structure analysis, the full set of 16,729 SNPs was used, as sNMF is robust to marker linkage. Population structure was assessed using sparse non-negative matrix factorization (sNMF) implemented in the R package LEA [87]. Cross-entropy was calculated for *K* = 2–6 (number of ancestral populations), with ten replicates per *K*. The *K* with the lowest cross-entropy was selected as optimal. The ancestry proportion matrix for *K* = 3 was visualized using ggplot2 [88]. A neighbor-joining (NJ) phylogenetic tree was constructed based on genetic dissimilarity calculated using the SNPRelate package [89] in R (v4.5.3). The kinship matrix was estimated using IBS analysis. Principal component analysis (PCA) was performed to visualize genetic variation.

### 4.4. Phenotypic Statistical Analysis

Statistical analysis was performed using Origin 2025b (v. 10.25) and GraphPad Prism (v. 9.5.1). Analysis of variance (ANOVA) was conducted to compare GPC across six environments, followed by Tukey’s honestly significant difference (HSD) test for multiple mean comparisons at *p* < 0.001. GPC was determined using a nitrogen analyzer (Kjeltec™8000MI, FOSS, Hilleroed, Denmark) (Table S7) [90]. Descriptive statistics, including the mean, standard deviation (SD), coefficient of variation (CV), minimum, and maximum values, were calculated for each environment. Pearson correlation analysis was used to assess relationships between environments and quality traits. All remaining statistical analyses were conducted in R (v4.5.3) using data.table and dplyr packages. Dendrogram construction and visualization utilized ape, dendextend, and ggdendro packages.

### 4.5. AMMI and Heritability Analysis

Additive main effects and multiplicative interaction (AMMI) analysis was performed on the genotype × year means matrix (99 genotypes × 3 years) from Yangma to partition GPC variation into genotype main effects (*αg*), environment main effects (*βe*), and G×E interaction effects, following the procedures described by Gauch (1988) [91] and Zobel et al. (1988) [92]. The interaction matrix was decomposed by singular value decomposition (SVD), and genotype stability was assessed using the coefficient of variation (CV) across years [93]. The AMMI model is represented as *Yge* = *μ* + *αg* + *βe* + *Σλnγgnδen* + *ρge*, where *Yge* is the phenotypic value, *μ* is the grand mean, *λn* is the singular value, *γgn* and *δen* are eigenvectors for genotype and environment, and *ρge* is the residual. All analyses were conducted in R v4.5.3 (R Core Team, 2025) [94].

Broad-sense heritability was estimated on an entry-mean basis using a linear mixed model fitted to averaged phenotypic values (mean of three within-plot replicates per environment as (GPC ~ Environment + (1|Genotype)) implemented in R v4.5.3 [94] with the lme4 package [47]. Entry-mean heritability was calculated as *H*^2^ = *Vg*/(*Vg* + *Ve*/*e*), where *Vg* is the genotypic variance, *Ve* is the residual variance, and e is the number of environments (*e* = 6).

### 4.6. Multi-Model Genome-Wide Association Mapping and Candidate Gene Identification Best Linear Unbiased Prediction (BLUP)

To account for genotype-by-environment (G×E) interaction and unequal replication across environments, BLUP values for GPC were calculated for each accession using a linear mixed model (GPC ~ Environment + (1|Genotype)) implemented in the lme4 package [47] in R (v4.5.3). Environment was fitted as a fixed effect and genotype as a random effect. BLUP values were extracted as the genotype random effect estimates plus the overall mean and used as the phenotypic input for multi-environment GWAS.

GWAS of GPC was conducted using phenotypic data from six environments (Yangma-2018, Yangma-2019, Deyang-2019, Nanchong-2019, Kangding-2020, and Yangma-2021) and GBS data from 266 HB accessions that passed stringent quality control.

Genotyping-by-sequencing (GBS) raw data were initially processed using the rMVP package (v.2.0) [95] in R (v.4.5.3). Quality control and genotype imputation were performed within rMVP, with filtering for MAF ≥ 0.05, resulting in 168,983 high-quality SNPs after removal of unmapped scaffolds (UN, *n* = 96). Association mapping was conducted on this full SNP set to maximize statistical power. Significance thresholds were established as follows: (i) a stringent Bonferroni-corrected genome-wide threshold at *p* < 2.95 × 10^−7^ (−log_10_(*p*) = 6.53), accounting for 168,983 independent tests; and (ii) a suggestive threshold at *p* < 1 × 10^−5^ (−log_10_(*p*) = 5.0) for exploratory associations.

Association mapping was conducted using three complementary models implemented in rMVP (v.2.0) [95]: (i) the general linear model (GLM), with population structure (Q matrix) fitted as fixed effects; (ii) the mixed linear model (MLM), which incorporates both population structure and kinship (*K* matrix) as random effects to control for cryptic relatedness; and (iii) FarmCPU, a multi-locus mixed model that iteratively optimizes the balance between polygenic back-ground and marker effects.

The GLM was specified as *y* = *Xβ* + *Qv* + *e***,** where *y* is the phenotypic vector, *X* is the genotypic matrix, *β* is the SNP effect vector, *Q* is the population structure matrix, *v* is the structure effect vector, and *e* is the residual error. The MLM was specified as *y* = *Xβ* + *Zu* + *e*, where *Z* is the kinship matrix, *u* is the random polygenic effect with *u* ~ *N*(0, *Kσ*^2^*g*), *K* is the identity-by-state (IBS) kinship matrix, and *σ*^2^*g* is the polygenic variance. FarmCPU was specified as *y* = *X_β_*+ X_β_+ e, iteratively optimizing between fixed-effect SNP testing and random-effect polygenic background estimation.

Cross-model validation was performed to identify robust association signals. SNPs detected at the suggestive threshold by two or more models (MLM, GLM, FarmCPU) in the BLUP analysis were considered cross-model stable. Additionally, cross-environment stability was assessed by identifying SNPs detected in multiple individual environments, with particular attention to loci showing consistent direction of effect across contrasting environments (Sichuan Basin vs. plateau). The genomic inflation factor (λ) was calculated for each model-environment combination to assess the adequacy of population structure correction, with λ values near 1.0 indicating appropriate control of false positives.

Computations were parallelized across four processing threads. Manhattan plots and quantile-quantile (QQ) plots were generated using the ggplot2 (v.3.5.1) and cowplot (v1.1.3) packages in R. Chromosome-wide cumulative positions were calculated to account for variable chromosome lengths, with alternating color schemes (blue/orange) applied per chromosome for visual clarity. Threshold lines were annotated directly on plots with Bonferroni (purple solid) and suggestive (red dashed) labels.

For functional interpretation, significant SNPs were anchored to the *Hordeum vulgare* Morex V3 reference genome [75] via WheatOmics (http://wheatomics.sdau.edu.cn/jbrowse.html, accessed on 8 June 2026). Candidate gene nomination for GPC proceeded through triangulation across the barley genome annotation (*HORVU.MOREX.r3* gene set), published functional studies on cereal grain protein metabolism, and expression validation using BarleyExp (http://barleyexp.com/common.html, accessed on 10 June 2026), a specialized repository of barley specific tissue RNA-seq data. Genes were prioritized based on functional relevance to nitrogen metabolism, amino acid transport, protein synthesis, or grain filling regulation, with expression in grain, seed, or senescence tissue serving as supporting evidence.

### 4.7. Linkage Disequilibrium (LD) Analysis

LD analysis was performed using the LDheatmap package [96] in R (v4.5.3). LD decay was assessed using pairwise *r*^2^ values calculated in TASSEL v5.2.90 [97] for intra-chromosomal SNP pairs within a 5Mb window. Due to strong population structure in this self-pollinated HB panel (PC1 = 53.6% variance; Figure 3), a schematic representation of LD decay was generated with a half-life of 0.75 Mb, consistent with published estimates for cultivated barley (0.02–4.3 Mb) [7,52]. The *r*^2^ = 0.2 threshold was used to define the boundary for candidate gene mining, corresponding to approximately ±2 Mb from lead SNPs.

## 5. Conclusions

This study addresses the crucial role of GPC in HB, a key quality trait for nutritional and industrial applications. To comprehensively understand the genetic factors governing GPC, we performed multi-environment GWAS across six environments: Yangma-2018, Yangma-2019, Deyang-2019, Nanchong-2019, Kangding-2020, and Yangma-2021. Phenotypic variation ranged from 5.02% to 18.31%, with moderate broad-sense heritability (*H*^2^ = 0.473) and significant G×E interaction. AMMI analysis confirmed that environment main effects accounted for 53% of total phenotypic variance, while genotype effects contributed only 18%, underscoring the necessity of multi-environment evaluation for reliable QTN detection. Genotyping-by-sequencing of 158 accessions yielded 168,983 SNPs, after stringent quality control. Multi-model GWAS (GLM, MLM, FarmCPU) identified 40 suggestive SNPs at *p* < 1 × 10^−5^, with chromosome 7H harboring the most robust cross-model stable locus (S7H_497728768/S7H_497728826). Two SNPs on chromosomes 6H (S6H_342117633, *p* = 1.99 × 10^−7^) and 7H (S7H_6966377, *p* = 1.55 × 10^−7^) exceeded the Bonferroni-corrected genome-wide significant threshold (*p* < 2.95 × 10^−7^) in the Yangma-2018 environment under GLM and FarmCPU. Candidate gene mining identified seven functional genes across four chromosomes, categorized into protein synthesis machinery (30S and 54S ribosomal proteins on 7H), nitrogen/amino acid metabolism (glutamate-cysteine ligase on 1H, GMP synthase on 6H, D-aminotransferase-like protein on 3H), and developmental regulation (seed maturation protein and tRNA dimethylallyltransferase on 7H and 3H). The lead SNP S7H_6966377 was located only 165 kb from the 30S ribosomal protein S13 gene, supporting a direct role in translational capacity during grain filling. The extended LD (half-life ~0.75 Mb) in this self-pollinated panel reflects strong population structure and supports the ±2 Mb candidate gene mining window. These findings establish 7H as a regulatory hub for GPC in HB and provide diagnostic markers for MAS in breeding programs. Fine-mapping and functional validation of the ribosomal protein and nitrogen metabolism candidates are warranted to confirm their causal roles and elucidate the regulatory networks connecting these genes to GPC variation.

## Figures and Tables

**Table 1:** Descriptive statistics of GPC (%) across six environments.

Location	*n*	Mean	Min.	Max.	SD	CV %	Skewness	Kurtosis
YM-2018	160	9.25 c	5.14	14.54	1.94	20.96	0.44	−0.01
YM-2019	144	11.00 b	5.13	15.39	2.00	18.21	−0.78	0.88
DY-2019	180	10.39 b	5.15	14.73	1.78	17.16	−0.37	0.38
NC-2019	149	10.68 b	5.02	18.31	2.37	22.21	0.59	1.12
KD-2020	186	11.55 a	6.04	16.87	2.02	17.53	−0.32	0.54
YM-2021	186	8.84 c	5.05	14.91	2.08	23.63	0.72	0.14

**Note:** Different letters denote significant differences between mean values at *p* < 0.001 (Tukey’s HSD test). *n*. = sample size, Min. = minimum, Max. = maximum, SD = Standard Deviation, CV = Coefficient of variation. **Environment codes:** YM-2018 = Yangma-2018; YM-2019 = Yangma-2019; DY-2019 = Deyang-2019; NC-2019 = Nanchong-2019; KD-2020 = Kangding-2020; YM-2021 = Yangma-2021.

**Table 2:** Correlation coefficient for GPC (%) across six environments.

	YM-2018	YM-2019	DY-2019	NC-2019	KD-2020	YM-2021
YM-2018	1					
YM-2019	0.83 ***	1				
DY-2019	0.94 ***	0.89 ***	1			
NC-2019	0.93 ***	0.91 ***	0.96 ***	1		
KD-2020	0.93 ***	0.90 ***	0.98 ***	0.97 ***	1	
YM-2021	0.94 ***	0.77 ***	0.90 ***	0.88 **	0.91 ***	1

**Note:** *** Indicates significant correlation at the 0.001 level (*p* < 0.001); ** indicates significant correlation at the 0.01 level (*p* < 0.01). **Environment codes:** YM-2018 = Yangma-2018; YM-2019 = Yangma-2019; DY-2019 = Deyang-2019; NC-2019 = Nanchong-2019; KD-2020 = Kangding-2020; YM-2021 = Yangma-2021.

**Table 3:** AMMI variance partitioning for GPC evaluated across three years at Yangma.

Source	df	SS	MS	% of Total SS	% of Interaction SS	Cumulative %
Genotype (G)	98	231.4	2.36	18	—	—
Environment (E)	2	680.21	340.1	53	—	—
G×E Interaction	196	372.63	1.9	29	100	—
IPC1	99	258.12	—	20.1	69.3	69.3
IPC2	97	114.51	—	8.9	30.7	100
Residual	0	0	—	0	0	100
Total	296	1284.24	—	100.0	—	—

**Table 4:** Summary statistics of GPC (%) for four quartile-based groups of 266 HB accessions across six environments.

GPC Group	*n*	Mean GPC (%)	SD	Min	Max	Mean SD Across Envs.	Mean CV (%)
High	46	11.68	0.48	11.07	13.39	2.01	17.2
Medium-High	45	10.72	0.18	10.42	11.05	1.94	18.1
Medium-Low	45	10.07	0.18	9.71	10.41	2.02	20.1
Low	46	8.91	0.8	5.66	9.7	2.02	22.7
Overall	182	10.29	1.17	5.66	13.39	2.00	19.4

***Note*:** GPC groups were defined by quartiles of mean GPC across available environments for 182 AMP-series accessions with multi-environment data: Low (≤Q1 = 9.70%), Medium-Low (Q1–Q2 = 9.71–10.41%), Medium-High (Q2–Q3 = 10.42–11.07%), High (>Q3 = 11.07%). SD = standard deviation; CV = coefficient of variation.

## Data Availability

The data presented in this study are available on request from the authors.

## Supplementary Materials

The following supporting information can be downloaded at: https://www.mdpi.com/article/10.3390/plants15152304/s1. Figure S1. Pearson correlation coefficients of GPC across six environments. Circle size and color intensity indicate correlation strength (*r*). Significance levels are indicated as *** *p* < 0.001 and ** *p* < 0.01; Figure S2. Mean GPC (%) across six environments. Error bars represent ±1 standard deviation. Environments are ordered by ascending mean GPC; Figure S3. KH-series accessions (*n* = 37), evaluated only at Nanchong-2019, were excluded from clustering due to limited environmental representation. Clustering was performed using Ward’s minimum variance method on Euclidean distances of *Z*-score standardized GPC values. Accessions were classified into four quartile-based groups: Low (dark blue, ≤9.70%, *n* = 46), Medium-Low (light blue, 9.71–10.41%, *n* = 45), Medium-High (orange, 10.42–11.07%, *n* = 45), and High (red, >11.07%, *n* = 46). Branch colors indicate the majority quartile group of descendant accessions. Tip labels are colored by their respective GPC group. Minor mixing between adjacent groups at branch boundaries reflects accessions with similar multi-environment GPC response patterns but different absolute GPC levels, indicating genotype-by-environment interactions; Figure S4. Manhattan plots for GPC analyzed using the mixed linear model (MLM) in six environments: (a) Yangma 2018, (b) Yangma 2019, (c) Deyang 2019, (d) Nanchong 2019, (e) Kangding 2020, and (f) Yangma 2021. The purple solid line indicates the Bonferroni-corrected genome-wide significance threshold (*p* < 2.95 × 10^−7^, −log_10_(*p*) = 6.53). The red dashed line indicates the suggestive significance threshold (*p* < 1 × 10^−5^, −log_10_(*p*) = 5.0). No SNP surpassed the Bonferroni threshold in any environment. Suggestive associations were detected in Yangma 2018 (4 SNPs), Yangma 2019 (3 SNPs), and Nanchong 2019 (1 SNP), while Deyang 2019, Kangding 2020, and Yangma 2021 showed no significant associations, reflecting strong genotype × environment interaction; Figure S5. Manhattan plots (left column) and quantile–quantile (QQ) plots (right column) comparing three association models for BLUP-derived GPC: (a,d) GLM, (b,e) MLM, and (c,f) FarmCPU. The purple solid line indicates the Bonferroni-corrected genome-wide significance threshold (*p* < 2.95 × 10^−7^, −log_10_(*p*) = 6.53). The red dashed line indicates the suggestive significance threshold (*p* < 1 × 10^−5^, −log_10_(*p*) = 5.0). A consistent suggestive locus on chromosome 7H (S7H_497728768 and S7H_497728826) was detected across all three models. Genomic inflation factors (λ) were 1.167 (GLM), 1.052 (MLM), and 1.167 (FarmCPU), indicating adequate control of population stratification in MLM and FarmCPU, while GLM showed moderate inflation consistent with uncorrected population structure; Table S1. Grain protein content (%) and best linear unbiased prediction (BLUP) values for hulless barley accessions across six environments. Values represent the means of three within-plot replicates per environment. The BLUP column shows genotype estimates integrated across all six environments; Table S2a. Phenotypic principal component scores of 182 hulless barley accessions across six environments; Table S2b. Genotypic principal component scores of hulless barley accessions based on GBS dataset.; Table S3. Complete list of stable SNPs associated with grain protein content detected across multiple environments and/or models in 243 hulless barley accessions. SNPs were identified by GWAS using GLM, MLM, and FarmCPU models across six environments. The search window was defined as ±2 Mb around each lead SNP; Table S4. Summary of genome-wide association analysis for grain protein content across six environments and combined BLUP using GLM, MLM, and FarmCPU models; Table S5. Candidate genes and their annotation within ±2 Mb of lead SNPs associated with grain protein content; Table S6. Information for 356 hulless barley accessions; Table S7. Kjeldahl nitrogen determination parameters for GPC.

## Abbreviations

The following abbreviations are used in this manuscript:

HB	Hulless barley
GPC	Grain protein content
SNP	Single nucleotide polymorphism
GWAS	Genome-wide association study
QTL	Quantitative trait locus
LD	Linkage disequilibrium
G×E	Genotype-by-environment
MAF	Minor allele frequency
MAS	Marker-assisted selection
PCA	Principal component analysis
BLUP	Best linear unbiased predictions
MLM	Mixed linear model
GLM	General linear model
AMMI	Additive main effects and multiplicative interaction

## References

[1] Yiblet Y., Misganaw W., Adamu E. (2024). Nutritional and Functional Composition of Barley Varieties from Legambo District, Ethiopia. Sci. World J..

[2] Middleton C.P., Stein N., Keller B., Kilian B., Wicker T. (2013). Comparative Analysis of Genome Composition in Triticeae Reveals Strong Variation in Transposable Element Dynamics and Nucleotide Diversity. Plant J..

[3] Harwood W.A. (2019). An Introduction to Barley: The Crop and the Model. Barley: Methods and Protocols.

[4] Romero C.C.T., Vels A., Niks R.E. (2018). Identification of a Large-Effect QTL Associated with Kernel Discoloration in Barley. J. Cereal Sci..

[5] Madakemohekar A.H., Prasad L.C., Lal J.P., Prasad R. (2018). Estimation of Combining Ability and Heterosis for Yield Contributing Traits in Exotic and Indigenous Crosses of Barley (*Hordeum vulgare* L.). Res. Crops.

[6] Haas M., Schreiber M., Mascher M. (2019). Domestication and Crop Evolution of Wheat and Barley: Genes, Genomics, and Future Directions. J. Integr. Plant Biol..

[7] Pasam R.K., Sharma R., Malosetti M., van Eeuwijk F.A., Haseneyer G., Kilian B., Graner A. (2012). Genome-Wide Association Studies for Agronomical Traits in a World Wide Spring Barley Collection. BMC Plant Biol..

[8] Eshghi R., Akhundova E. (2010). Genetic Diversity in Hulless Barley Based on Agromorphological Traits and RAPD Markers and Comparison with Storage Protein Analysis. Afr. J. Agric. Res..

[9] Dondup D., Yang Y., Xu D., Namgyal L., Wang Z., Shen X., Dorji T., Kyi N., Drolma L., Gao L. (2023). Genome Diversity and Highland-Adaptative Variation in Tibet Barley Landrace Population of China. Front. Plant Sci..

[10] Zeng Y., Pu X., Yang J., Du J., Yang X., Li X., Li L., Zhou Y., Yang T. (2018). Preventive and Therapeutic Role of Functional Ingredients of Barley Grass for Chronic Diseases in Human Beings. Oxidative Med. Cell. Longev..

[11] Zhou X., Yu J., Spengler R.N., Shen H., Zhao K., Ge J., Bao Y., Liu J., Yang Q., Chen G. (2020). 5200-Year-Old Cereal Grains from the Eastern Altai Mountains Redate the Trans-Eurasian Crop Exchange. Nat. Plants.

[12] Dyulgerov N., Dyulgerova B. (2024). Grain Yield and Yield-Related Traits of Hulled and Hull-Less Spring. Bulg. J. Agric. Sci..

[13] Ferreira J.R., Pereira J.F., Turchetto C., Minella E., Consoli L., Delatorre C.A. (2016). Assessment of Genetic Diversity in Brazilian Barley Using SSR Markers. Genet. Mol. Biol..

[14] Liang L., Zhong X., Li L., Jia J., Ma L., Wang S., Tu R., Liu Y., Feng Z., Luo H. (2025). Impact of Regional Variability on the Flavor Profile of Chinese Hulless Barley Wines. LWT.

[15] Patial M., Thakur V., Bishnoi S.K. (2024). Hulless Barley: A Nutritional Powerhouse. Vigyan Varta.

[16] Zeng X., Long H., Wang Z., Zhao S., Tang Y., Huang Z., Wang Y., Xu Q., Mao L., Deng G. (2015). The Draft Genome of Tibetan Hulless Barley Reveals Adaptive Patterns to the High Stressful Tibetan Plateau. Proc. Natl. Acad. Sci. USA.

[17] Kumbhar R.A., Yang K., Min T., Memon S., Benhafid R.C., Jiale J., Mari K., Cao Y., Chen W., Liu Y. (2026). Genetic Exploration of β-Glucan Content in Barley Grains through GWAS and RNA-Sequencing Approaches. Mol. Breed..

[18] An L., Wang Z., Cui Y., Bai Y., Yao Y., Yao X., Wu K. (2024). Comparative Analysis of Hulless Barley Transcriptomes to Regulatory Effects of Phosphorous Deficiency. Life.

[19] Liu B., Ma G., Bussmann R.W., Bai K., Li J., Cao W., Long C. (2019). Determining Factors for the Diversity of Hulless Barley Agroecosystem in the Himalaya Region—A Case Study from Northwest Yunnan, China. Glob. Ecol. Conserv..

[20] Yao X., Wu K., Yao Y., Bai Y., Ye J., Chi D. (2018). Construction of a High-Density Genetic Map: Genotyping by Sequencing (GBS) to Map Purple Seed Coat Color (Psc) in Hulless Barley. Hereditas.

[21] Kumar V., Khippal A., Singh J., Selvakumar R., Malik R., Kumar D., Kharub A.S., Verma R.P.S., Sharma S. (2014). Developing Selection Criteria Based on an Ontogenetic Path Analysis Approach to Improve Grain Yield in Barley. Ournal Wheat Res..

[22] Matsumoto T., Tanaka T., Sakai H., Amano N., Kanamori H., Kurita K., Kikuta A., Kamiya K., Yamamoto M., Ikawa H. (2011). Comprehensive Sequence Analysis of 24,783 Barley Full-Length CDNAs Derived from 12 Clone Libraries. Plant Physiol..

[23] Geng L., Li M., Zhang G., Ye L. (2022). Barley: A Potential Cereal for Producing Healthy and Functional Foods. Food Qual. Saf..

[24] Martre P., Jamieson P.D., Semenov M.A., Zyskowski R.F., Porter J.R., Triboi E. (2006). Modelling Protein Content and Composition in Relation to Crop Nitrogen Dynamics for Wheat. Eur. J. Agron..

[25] Sha A., Liu B., Liu C., Sun Q., Chen M., Peng L., Zou L., Zhao C., Li Q. (2024). Highland Barley ELNs and Physiological Responses to Different Concentrations of Cr (VI) Stress. Ecotoxicol. Environ. Saf..

[26] Tanner G.J., Colgrave M.L., Blundell M.J., Howitt C.A., Bacic A. (2019). Hordein Accumulation in Developing Barley Grains. Front. Plant Sci..

[27] Gous P.W., Gilbert R.G., Fox G.P. (2015). Drought-Proofing Barley (*Hordeum vulgare*) and Its Impact on Grain Quality: A Review. J. Inst. Brew..

[28] Kumbhar R.A., Memon S., Hussain M., Liu Y., Feng Z., Zhao H. (2026). Unveiling the Potential of Functional Components in Hull-Less Barley Grains: Health Benefits, Structural Composition, and Genetic Advancements. Foods.

[29] Fan C., Zhai H., Wang H., Yue Y., Zhang M., Li J., Wen S., Guo G., Zeng Y., Ni Z. (2017). Identification of QTLs Controlling Grain Protein Concentration Using a High-Density SNP and SSR Linkage Map in Barley (*Hordeum vulgare* L.). BMC Plant Biol..

[30] Šterna V., Zute S., Jansone I., Kantane I. (2017). Chemical Composition of Covered and Naked Spring Barley Varieties and Their Potential for Food Production. Pol. J. Food Nutr. Sci..

[31] Li X., Yu X., Yao X., Yao Y., Bai Y., An L., Wang H., Wu K. (2022). Developmental Stages of Young Spikes in Six-Rowed Qingke (*Hordeum vulgare* L.). Plant Growth Regul..

[32] Zhang L., Miao L., He J., Li H., Li M. (2022). The Transcriptome and Metabolome Reveal the Potential Mechanism of Lodging Resistance in Intergeneric Hybrids between Brassica Napus and Capsella Bursa-Pastoris. Int. J. Mol. Sci..

[33] Baik B.-K. (2014). Processing of Barley Grain for Food and Feed. Barley.

[34] Kumar D., Narwal S., Kharub A.S., Singh G.P. (2019). Scope of Food Barley Research and Development in India. Wheat Barley Res..

[35] Jaeger A., Zannini E., Sahin A.W., Arendt E.K. (2021). Barley Protein Properties, Extraction and Applications, with a Focus on Brewers’ Spent Grain Protein. Foods.

[36] Garcia-Valle D.E., Bello-Pérez L.A., Agama-Acevedo E., Tovar J., Aguirre-Cruz A., Alvarez-Ramirez J. (2022). Effect of the Preparation Method on Structural and In Vitro Digestibility Properties of Type II Resistant Starch-Enriched Wheat Semolina Pasta. J. Cereal Sci..

[37] Senarathna S., Mel R., Malalgoda M. (2024). Utilization of Cereal-Based Protein Ingredients in Food Applications. J. Cereal Sci..

[38] Narwal S., Kumar D., Sheoran S., Verma R.P.S., Gupta R.K. (2017). Hulless Barley as a Promising Source to Improve the Nutritional Quality of Wheat Products. J. Food Sci. Technol..

[39] Bleidere M., Jansone Z., Grunte I., Jakobsone I. (2017). Biochemical Composition of Spring Barley Grain Pearled to Varying Degrees. Proc. Latv. Acad. Sci. Sect. B Nat. Exact Appl. Sci..

[40] Farag M.A., Xiao J., Abdallah H.M. (2022). Nutritional Value of Barley Cereal and Better Opportunities for Its Processing as a Value-Added Food: A Comprehensive Review. Crit. Rev. Food Sci. Nutr..

[41] Meng G., Rasmussen S.K., Christensen C.S.L., Fan W., Torp A.M. (2023). Molecular Breeding of Barley for Quality Traits and Resilience to Climate Change. Front. Genet..

[42] Vaishnavi A.S., Mamta, Sandal A. (2024). Physical and Nutritional Evaluation of Hulled and Hulless Barley. Int. J. Adv. Biochem. Res..

[43] Ross A.S., Meints B.M., Bunting J.S. (2023). Barley. ICC Handbook of 21st Century Cereal Science and Technology.

[44] South Dakota State University, Cooperative Extension (1980). Barley Proteins.

[45] Baik B.-K., Ullrich S.E. (2008). Barley for Food: Characteristics, Improvement, and Renewed Interest. J. Cereal Sci..

[46] Fox G.P. (2009). Beer and Arabinoxylan. Beer in Health and Disease Prevention.

[47] Bates D., Mächler M., Bolker B., Walker S. (2015). Fitting Linear Mixed-Effects Models Using Lme4. J. Stat. Softw..

[48] Gayacharan, Joshi D.C., Aravind J., Wankhede D.P., Singh B., Kumar P., Rajkumar S., Parida S.K., Semwal D.P., Sharma P. (2026). Multi-Environment Phenotyping of Ricebean (*Vigna umbellata* (Thunb.) Ohwi & Ohashi) Germplasm and Identification of Core Set for Accelerating the Crop Improvement Programs. Front. Plant Sci..

[49] Skovbjerg C.K., Sarup P., Wahlström E., Jensen J.D., Orabi J., Olesen L., Jensen J., Jahoor A., Ramstein G. (2025). Correction: Multi-Population GWAS Detects Robust Marker Associations in a Newly Established Six-Rowed Winter Barley Breeding Program. Heredity.

[50] Boehm J.D., Nguyen V., Tashiro R.M., Anderson D., Shi C., Wu X., Woodrow L., Yu K., Cui Y., Li Z. (2018). Genetic Mapping and Validation of the Loci Controlling 7S A′ and 11S A-Type Storage Protein Subunits in Soybean [*Glycine max* (L.) Merr.]. Theor. Appl. Genet..

[51] Mickelson S., See D., Meyer F.D., Garner J.P., Foster C.R., Blake T.K., Fischer A.M. (2003). Mapping of QTL Associated with Nitrogen Storage and Remobilization in Barley (*Hordeum vulgare* L.) Leaves. J. Exp. Bot..

[52] Caldwell K.S., Russell J., Langridge P., Powell W. (2006). Extreme Population-Dependent Linkage Disequilibrium Detected in an Inbreeding Plant Species, Hordeum Vulgare. Genetics.

[53] Bengtsson T., Manninen O., Jahoor A., Orabi J., The PPP Barley Consortium (2017). Genetic Diversity, Population Structure and Linkage Disequilibrium in Nordic Spring Barley (*Hordeum vulgare* L. subsp. *vulgare*). Genet. Resour. Crop Evol..

[54] Genievskaya Y., Almerekova S., Abugalieva S., Chudinov V., Blake T., Abugalieva A., Turuspekov Y. (2022). Identification of SNP Markers Associated with Grain Quality Traits in a Barley Collection (*Hordeum vulgare* L.) Harvested in Kazakhstan. Agronomy.

[55] Kaczmarek Z., Adamski T., Surma M., Jezowski S., Leśniewska-Frątczak M. (1999). Genotype–Environment Interaction of Barley Doubled Haploids with Regard to Malting Quality. Plant Breed..

[56] Wang Y., Ren X., Sun D., Sun G. (2015). Origin of Worldwide Cultivated Barley Revealed by NAM-1 Gene and Grain Protein Content. Front. Plant Sci..

[57] Kaur S., Kaur H., Singh P., Cheema B.S., Kumar V. (2016). Genetic Variation and Evaluation of Exotic Barley (*Hordeum vulgare* L.) Genotypes for Grain Protein Content, Starch Content and Agronomic Traits. Electron. J. Plant Breed..

[58] Emebiri L.C., Moody D.B., Horsley R., Panozzo J., Read B.J. (2005). The Genetic Control of Grain Protein Content Variation in a Doubled Haploid Population Derived from a Cross between Australian and North American Two-Rowed Barley Lines. J. Cereal Sci..

[59] Cai S., Yu G., Chen X., Huang Y., Jiang X., Zhang G., Jin X. (2013). Grain Protein Content Variation and Its Association Analysis in Barley. BMC Plant Biol..

[60] Luan H., Gao J., Qu X., Li Y., Wu Y., Wang J., Li S., Xu M., Xu X., Sun M. (2025). Integrating Genome-Wide Association and Whole Transcriptome Analysis to Reveal Genetic Control of Grain Quality Traits in Barley. Euphytica.

[61] Pour-Aboughadareh A., Koohkan S., Omrani A., Marzooghian A., Gholipour A., Zali H., Kheirgoo M., Shahbazi-Homonloo K., Poczai P., Jamshidi B. (2025). Combining Multiple Stability and Adaptation Models to Analyze Genotype-by-environment Interactions for Selection of Stable Barley Genotypes with High Yield Performance. Agrosyst. Geosci. Environ..

[62] Bonfil D.J., Abbo S., Degen D., Simchon Y., Ben-David R. (2023). Towards Stable Wheat Grain Yield and Quality under Climatic Instability. Agron. J..

[63] Greveniotis V., Bouloumpasi E., Skendi A., Zotis S., Kantas D., Ipsilandis C.G. (2026). Grain Quality and Stability of Advanced Barley Lines and Local Landraces in Mediterranean Conditions. Agriculture.

[64] Akdemir D., Beavis W., Fritsche-Neto R., Singh A.K., Isidro-Sánchez J. (2019). Multi-Objective Optimized Genomic Breeding Strategies for Sustainable Food Improvement. Heredity.

[65] Lama S. (2020). Bread-Making Quality in a Changing Climate: In Search of Climate Stable Genotypes and Robust Screening Methods for Wheat.

[66] Malosetti M., Ribaut J.M., van Eeuwijk F.A. (2013). The Statistical Analysis of Multi-Environment Data: Modeling Genotype-by-Environment Interaction and Its Genetic Basis. Front. Plant Sci..

[67] Crossa J., Pérez-Rodríguez P., Cuevas J., Montesinos-López O., Jarquín D., de los Campos G., Burgueño J., González-Camacho J.M., Pérez-Elizalde S., Beyene Y. (2017). Genomic Selection in Plant Breeding: Methods, Models, and Perspectives. Trends Plant Sci..

[68] Yan W., Kang M.S. (2002). GGE Biplot Analysis.

[69] Price A.L., Patterson N.J., Plenge R.M., Weinblatt M.E., Shadick N.A., Reich D. (2006). Principal Components Analysis Corrects for Stratification in Genome-Wide Association Studies. Nat. Genet..

[70] Zhang Z., Ersoz E., Lai C.-Q., Todhunter R.J., Tiwari H.K., Gore M.A., Bradbury P.J., Yu J., Arnett D.K., Ordovas J.M. (2010). Mixed Linear Model Approach Adapted for Genome-Wide Association Studies. Nat. Genet..

[71] Yu J., Pressoir G., Briggs W.H., Vroh Bi I., Yamasaki M., Doebley J.F., McMullen M.D., Gaut B.S., Nielsen D.M., Holland J.B. (2006). A Unified Mixed-Model Method for Association Mapping That Accounts for Multiple Levels of Relatedness. Nat. Genet..

[72] Liu X., Huang M., Fan B., Buckler E.S., Zhang Z. (2016). Iterative Usage of Fixed and Random Effect Models for Powerful and Efficient Genome-Wide Association Studies. PLoS Genet..

[73] Jiang C., Kan J., Gao G., Dockter C., Li C., Wu W., Yang P., Stein N. (2025). Barley2035: A Decadal Vision for Barley Research and Breeding. Mol. Plant.

[74] Chen W., Gao Y., Xie W., Gong L., Lu K., Wang W., Li Y., Liu X., Zhang H., Dong H. (2014). Genome-Wide Association Analyses Provide Genetic and Biochemical Insights into Natural Variation in Rice Metabolism. Nat. Genet..

[75] Mascher M., Gundlach H., Himmelbach A., Beier S., Twardziok S.O., Wicker T., Radchuk V., Dockter C., Hedley P.E., Russell J. (2017). A Chromosome Conformation Capture Ordered Sequence of the Barley Genome. Nature.

[76] Jia Y., Westcott S., He T., McFawn L.A., Angessa T., Hill C., Tan C., Zhang X., Zhou G., Li C. (2021). Genome-Wide Association Studies Reveal QTL Hotspots for Grain Brightness and Black Point Traits in Barley. Crop J..

[77] Zhou Q., Xi B., Shoaib N., Gao Y., Cheng Z., Kumbhar R.A., Feng Z., Liu Y., Zhao H., Yu G. (2024). Preparation of Polyclonal Antibodies to Barley Granule-Bound Amylopectin Synthase Ia and Their Application in the Characterization of Interacting Proteins. Agronomy.

[78] Battenfield S. (2015). Genomic Selection and Association Mapping for Wheat Processing and End-Use Quality.

[79] Cockram J., White J., Zuluaga D.L., Smith D., Comadran J., Macaulay M., Luo Z., Kearsey M.J., Werner P., Harrap D. (2010). Genome-Wide Association Mapping to Candidate Polymorphism Resolution in the Unsequenced Barley Genome. Proc. Natl. Acad. Sci. USA.

[80] Kumbhar R.A., Yang K., Mari Baloch S.N., Memon S., Liu Y., Zhao H., Feng Z. (2025). Exploring the Significance of β-Glucan in Grains of Hulless Barley. Plant Mol. Biol. Rep..

[81] Moin M., Bakshi A., Saha A., Dutta M., Madhav S.M., Kirti P.B. (2016). Rice Ribosomal Protein Large Subunit Genes and Their Spatio-Temporal and Stress Regulation. Front. Plant Sci..

[82] Xu L., Wang P., Zhang X., Zhang Q., Wang P., Xu Y., Li C., Zhang W. (2025). Natural Variations in a Barley Aldehyde Oxidase 1 Gene Affect Seed Germination and Malting Quality. Crop J..

[83] Bahrami F., Arzani A., Rahimmalek M., Araniti F. (2025). Transcriptome Alterations Related to Heat Stress Responses of Wild and Cultivated Barley. Plant Physiol. Biochem..

[84] Wang G., Wang J., Yao L., Li B., Ma X., Si E., Yang K., Li C., Shang X., Meng Y. (2023). Transcriptome and Metabolome Reveal the Molecular Mechanism of Barley Genotypes Underlying the Response to Low Nitrogen and Resupply. Int. J. Mol. Sci..

[85] Elshire R.J., Glaubitz J.C., Sun Q., Poland J.A., Kawamoto K., Buckler E.S., Mitchell S.E. (2011). A Robust, Simple Genotyping-by-Sequencing (GBS) Approach for High Diversity Species. PLoS ONE.

[86] Ward J.H. (1963). Hierarchical Grouping to Optimize an Objective Function. J. Am. Stat. Assoc..

[87] Frichot E., François O. (2015). LEA: An R Package for Landscape and Ecological Association Studies. Methods Ecol. Evol..

[88] Wickham H. (2016). Ggplot2: Elegant Graphics for Data Analysis.

[89] Zheng X., Levine D., Shen J., Gogarten S.M., Laurie C., Weir B.S. (2012). A High-Performance Computing Toolset for Relatedness and Principal Component Analysis of SNP Data. Bioinformatics.

[90] Yu W., Tan X., Zou W., Hu Z., Fox G.P., Gidley M.J., Gilbert R.G. (2017). Relationships between Protein Content, Starch Molecular Structure and Grain Size in Barley. Carbohydr. Polym..

[91] Gauch H.G. (1988). Model Selection and Validation for Yield Trials with Interaction. Biometrics.

[92] Zobel R.W., Wright M.J., Gauch H.G. (1988). Statistical Analysis of a Yield Trial. Agron. J..

[93] Francis T.R., Kannenberg L.W. (1978). Yield Stability Studies in Short-Season Maize. I. A Descriptive Method for Grouping Genotypes. Can. J. Plant Sci..

[94] R Foundation for Statistical Computing (2025). R Core Team R: A Language and Environment for Statistical Computing.

[95] Yin L., Zhang H., Tang Z., Xu J., Yin D., Zhang Z., Yuan X., Zhu M., Zhao S., Li X. (2021). RMVP: A Memory-Efficient, Visualization-Enhanced, and Parallel-Accelerated Tool for Genome-Wide Association Study. Genom. Proteom. Bioinform..

[96] Shin J.-H., Blay S., Graham J., McNeney B. (2006). LDheatmap: An R Function for Graphical Display of Pairwise Linkage Disequilibria Between Single Nucleotide Polymorphisms. J. Stat. Softw..

[97] Bradbury P.J., Zhang Z., Kroon D.E., Casstevens T.M., Ramdoss Y., Buckler E.S. (2007). TASSEL: Software for Association Mapping of Complex Traits in Diverse Samples. Bioinformatics.

